# The cholinergic basal forebrain in the ferret and its inputs to the auditory cortex

**DOI:** 10.1111/ejn.12653

**Published:** 2014-06-19

**Authors:** Victoria M Bajo, Nicholas D Leach, Patricia M Cordery, Fernando R Nodal, Andrew J King

**Affiliations:** Department of Physiology, Anatomy and Genetics, University of OxfordSherrington Building, Parks Road, Oxford, OX1 3PT, UK

**Keywords:** acetylcholine, immunocytochemistry, immunotoxin, neuromodulation, nucleus basalis, p75 neurotrophin receptor

## Abstract

Cholinergic inputs to the auditory cortex can modulate sensory processing and regulate stimulus-specific plasticity according to the behavioural state of the subject. In order to understand how acetylcholine achieves this, it is essential to elucidate the circuitry by which cholinergic inputs influence the cortex. In this study, we described the distribution of cholinergic neurons in the basal forebrain and their inputs to the auditory cortex of the ferret, a species used increasingly in studies of auditory learning and plasticity. Cholinergic neurons in the basal forebrain, visualized by choline acetyltransferase and p75 neurotrophin receptor immunocytochemistry, were distributed through the medial septum, diagonal band of Broca, and nucleus basalis magnocellularis. Epipial tracer deposits and injections of the immunotoxin ME20.4-SAP (monoclonal antibody specific for the p75 neurotrophin receptor conjugated to saporin) in the auditory cortex showed that cholinergic inputs originate almost exclusively in the ipsilateral nucleus basalis. Moreover, tracer injections in the nucleus basalis revealed a pattern of labelled fibres and terminal fields that resembled acetylcholinesterase fibre staining in the auditory cortex, with the heaviest labelling in layers II/III and in the infragranular layers. Labelled fibres with small *en-passant* varicosities and simple terminal swellings were observed throughout all auditory cortical regions. The widespread distribution of cholinergic inputs from the nucleus basalis to both primary and higher level areas of the auditory cortex suggests that acetylcholine is likely to be involved in modulating many aspects of auditory processing.

## Introduction

The central cholinergic system has been characterized in a number of animal species, including rodents and primates (Mesulam *et al*., 1984; Saper, 1984; Woolf, 1991). The basal forebrain (BF) cholinergic complex, encompassing the medial septum (MS), horizontal and vertical limbs of the diagonal band of Broca (HDB and VDB, respectively) and nucleus basalis magnocellularis (NB), provides the majority of the cholinergic innervation to the sensory, motor and prefrontal cortices and to the hippocampus (Semba & Fibiger, 1989). Of these BF regions, the NB provides the majority of the cholinergic input to the sensory cortices (Saper, 1984; Henderson, 1987a; Kamke *et al*., 2005a).

The NB receives its inputs mainly from limbic regions, including the nucleus accumbens, amygdala and hypothalamus, as well as from higher cortical sensory areas (Mesulam & Mufson, 1984) and the prefrontal cortex (Rasmusson *et al*., 2007). In addition, prominent projections from the brainstem to the NB, mainly from the pontomesencephalic tegmentum, including the substantia nigra pars compacta, raphe nuclei, pedunculopontine tegmental nucleus, retrorubral area and ventral tegmental area, have been described in the monkey (Russchen *et al*., 1985) and in rodents (Semba *et al*., 1988; Jones & Cuello, 1989). It therefore appears well placed to relay signals about the salience of sensory stimuli directly to the cortex, and represents an obvious target for manipulation to recreate those increases in cortical cholinergic efflux observed when animals are actively engaged in a behavioural task (Rasmusson & Szerb, 1976; Dalley *et al*., 2001).

The importance of cortical cholinergic afferents in enhancing the representation of behaviourally-relevant stimuli has long been recognized. For example, pairing sounds with electrical stimulation of the NB results in stimulus-specific plasticity in the auditory cortex that closely resembles the changes observed in animals that have undergone classical or operant conditioning (Kilgard & Merzenich, 1998a,b1998b, 2002; Weinberger & Bakin, 1998; Miasnikov *et al*., 2001; Ma & Suga, 2003; Froemke *et al*., 2007; Reed *et al*., 2011).

In recent years, the ferret has become one of the main animal models in auditory neuroscience. Rapid task-dependent plasticity has been observed in the primary auditory cortex (A1) of this species, which is thought to enhance the representation of target stimuli relative to that of background sounds (Fritz *et al*., 2007; Atiani *et al*., 2009). Moreover, the ferret auditory cortex plays a critical role in learning-induced behavioural plasticity (Bajo *et al*., 2010; Nodal *et al*., 2012) and cholinergic inputs to the auditory cortex have recently been shown to be involved in this (Leach *et al*., 2013). In order to understand the mechanisms underlying the effects of attention and learning on auditory processing, it is essential to identify the circuitry by which cholinergic forebrain neurons influence the auditory cortex. However, in contrast to other species, particularly rodents (Saper, 1984; Grove, 1988; Gyengesi *et al*., 2013; Zaborszky *et al*., 2013), very little anatomical data are available in ferrets.

In this study, we used a variety of complementary methods to characterize the organization of the cholinergic BF and its projections to the auditory cortex of the ferret, paving the way for future work on the role of these circuits in cortical processing and plasticity.

## Materials and methods

Fourteen adult ferrets (*Mustela putorius furo*) of both sexes were used in this study. All experiments were approved by a local Ethical Review Committee at the University of Oxford, and performed under licence from the UK Home Office in accordance with the Animal (Scientific Procedures) Act 1986.

**Figure 1:**
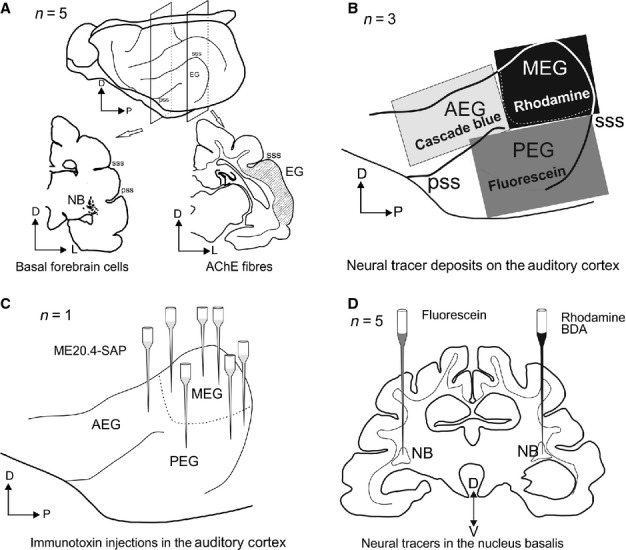
Four approaches were used to characterize the cholinergic BF and its connections to the auditory cortex. (A) Identification of fibres stained for AChE in the auditory cortex and cholinergic cell bodies in the BF by ChAT and p75^NTR^ immunocytochemistry. (B) Epipial deposits of fluorescent tracers on the auditory cortex. (C) Multiple injections of the immunotoxin ME20.4-SAP in the auditory cortex. (D) Bilateral fluorescent tracer injections in the NB. AChE, acetylcholinesterase; AEG, anterior ectosylvian gyrus; BDA, biotinylated dextran amine; D, dorsal; EG, ectosylvian gyrus; L, lateral; ME20.4-SAP, monoclonal antibody specific for the p75^NTR^ conjugated to saporin; MEG, middle ectosylvian gyrus; NB, nucleus basalis; P, posterior; PEG, posterior ectosylvian gyrus; pss, pseudosylvian sulcus; sss, suprasylvian sulcus; V, ventral.

Figure1 summarizes the different experimental approaches used in this study and the number of animals included for each experimental procedure. Five animals were used to determine the distribution of cholinergic and inhibitory neurons in the BF and of acetylcholinesterase (AChE) fibres in the auditory cortex (Fig.1A). The connections between these brain regions were identified by making epipial neural tracer deposits on the surface of the auditory cortex (Fig.1B, *n* = 3), by evaluation of cell loss in the NB after multiple injections of the cholinergic immunotoxin ME20.4-SAP were made in the auditory cortex (Fig.1C, *n* = 1), and by injecting neural tracers into the NB (Fig.1D, *n* = 5).

### Cholinergic neurons in the basal forebrain and acetylcholinesterase fibres in the auditory cortex

An initial group of three animals were sedated with Domitor (medetomidine hydrochloride; 0.1 mg/kg body weight i.m., Pfizer Ltd, Kent, UK), overdosed with Euthatal (2 mL of 200 mg/mL i.p., pentobarbitone sodium; Merial Animal Health Ltd, Harlow, UK) and transcardially perfused with 300 mL of 0.9% saline at room temperature (22 °C), followed by 1 L of 4% paraformaldehyde in 0.1 m phosphate buffer (PB), pH 7.4. After removal from the skull, brains were cryoprotected by immersion in 30% (w/v) sucrose in 0.1 m PB with agitation until sunk. Each brain was mounted onto a freezing stage (Physitemp Instruments Inc., Clifton, NJ, USA) and sectioned in the coronal plane at a thickness of 35 μm, from rostral to caudal, using a microtome (SM200R; Leica Microsystems UK Ltd, Heerbrugg, Switzerland). Sections were collected in 0.1 m PB in eight sequential series, each of which followed a different staining protocol.

Nissl substance was stained in the first set of serial sections with 0.5% cresyl violet and myelin was stained according to the method of Gallyas (1979) in another set, whereas two other non-contiguous sets were processed for AChE histochemistry using the protocol described in Kamke *et al*. (2005a) (modified from Tago *et al*., 1986). Finally, antibodies (Table1) against non-phosphorylated neurofilament H (SMI_32_; Covance Research Products Inc., Emeryville, CA, USA), choline acetyltransferease (ChAT; Millipore Corporation, Temecula, CA, USA and Advanced Targeting Systems, San Diego, CA, USA) and p75^NTR^ (Advanced Targeting Systems) were used separately in three further sets of sections.

**Table 1:** Summary of primary antibodies

Antigen	Immunogen	Manufacturing details (including animal raised in)	Working dilution
SMI_32_	Rat hypothalamus homogenate	Covance Research Products Inc. (Emeryville, CA, USA), mouse monoclonal, cat. no. SMI32R	1 : 1000
ChAT	Human placental enzyme ChAT	Millipore Corporation (Temecula, CA, USA), rabbit polyclonal, cat. no. AB143	1 : 1000
ChAT	22 amino-acid peptide from porcine ChAT coupled to KLH	Advanced Targeting Systems (San Diego, CA, USA), rabbit polyclonal, cat. no. AB-N34ap	1 : 1000
p75^NTR^	Clon ME20.4, WM245 melanoma cells	Advanced Targeting Systems, mouse monoclonal, cat. no. AB-N07	1 : 500
GAD67	Recombinant fragment (region within AA 5 and 176 of GAD67)	Abcam plc (Cambridge, UK), rabbit polyclonal, cat. no. ab97739	1 : 100
Parvalbumin	Frog muscle parvalbumin (clone PARV-19)	Sigma–Aldrich (Dorset, UK), mouse monoclonal, cat. no. P3088	1 : 6000
Green fluorescent protein (GFP)	GFP-tagged fusion protein (clone GFP-20)	Sigma–Aldrich (Dorset, UK), mouse monoclonal, cat. no. G6539	1 : 6000
Tetramethyl-rhodamine dextran	Tetramethyl-rhodamine	Molecular Probes Inc. (Eugene, OR, USA), rabbit polyclonal, IgG fraction, cat. no. A6397	1 : 6000
Fluorescein dextran	Fluorescein/Oregon green	Molecular Probes Inc., rabbit polyclonal, IgG fraction, cat. no. A889	1 : 6000
Cascade blue	Alexa Fluor 405/cascade blue	Molecular Probes Inc., rabbit polyclonal IgG fraction, cat. no. A5760	1 : 6000

The AChE histochemistry was carried out on free-floating sections with an initial incubation in 0.1% H_2_O_2_ in 0.1 m phosphate-buffered saline (PBS), pH 7.4, followed by washes in 0.1 m acetate/acetic acid buffer, pH 6.0. Sections were then preincubated in 10^−5^ m tetraisopropyl pyrophosophoramide (Sigma–Aldrich Company Ltd) for 120 min at 37 °C and, after further washes in 0.1 m acetate/acetic acid buffer, incubated in 250 μm sodium citrate, 150 μm copper sulphate, 25 μm potassium ferricyanide and 125 μm acetylthiocholine iodide for 30 min at room temperature. Finally, sections were reacted in 0.04% 3,3′-diaminobenzidine tetrahydrochloride (DAB; Sigma–Aldrich) + 0.3% ammonium nickel sulphate in Tris buffer for 5 min and then for a further 12 min with the addition of 0.003% H_2_O_2_.

When SMI_32_, anti-ChAT and anti-p75^NTR^ immunocytochemistry was carried out, sections were first incubated in PBS with 5% normal horse serum for 1 h to reduce non-specific binding of the antibody and then in the corresponding primary antibody with 3% horse serum for 72 h at 5 °C. The ChAT antibodies were diluted in PBS with 0.1% Triton X_100_, whereas SMI_32_ and p75^NTR^ antibodies were diluted in PBS alone. Biotinylated secondary antibodies (horse anti-rabbit for ChAT and horse anti-mouse for SMI_32_ and p75^NTR^; Vector Laboratories, Burlingame, CA, USA) were used at a 1 : 200 dilution with 3% horse serum in PBS for 2 h, followed by 90 min incubation with an avidin–biotin complex (Vectastain Elite ABC; Vector Laboratories). The immunostaining was visualized using DAB with nickel–cobalt intensification (Adams, 1981) for SMI_32_ detection and DAB alone for p75^NTR^ and ChAT detection (0.4 mm DAB and 9.14 mm H_2_O_2_ in 0.1 m PB until the reaction product was visualized). All sections were mounted onto slides, dried, dehydrated with absolute ethanol, cleared with xylene and coverslipped with DePeX (Merck Chemicals Ltd, Nottingham, UK).

Two additional animals were used for double immunoreactions to analyse ChAT and anti-p75^NTR^ co-localization and to visualize putative inhibitory neurons in the BF. In a set of serial sections (one in six), fluorescent-labelled secondary antibodies (anti-rabbit conjugated to Alexa Fluor® 488 for ChAT and anti-mouse conjugated to Alexa Fluor® 568; 1 : 200, 2 h; Molecular Probes Inc., Eugene, OR, USA) were used together after the corresponding primary antibodies were included in a first incubation (the same antibodies and dilutions as previously mentioned, see also Table1). After the secondary incubation, the sections were rinsed in PBS, mounted and coverslipped using a non-permanent mounting medium for fluorescence (Vectashield; Vector Laboratories). Visualization of putative inhibitory neurons was performed with double immunostaining by pairing p75^NTR^ with Pv or 67 kDa glutamic acid decarboxylase (GAD67) antibodies (Table1) in one in every six sections at the level of the NB. Differential immunostaining was subsequently visualized using DAB (brown reaction product) for p75^NTR^ and Vector VIP (violet reaction product; Vector VIP peroxidase substrate kit, Vector Laboratories) for Pv or GAD67, or by performing two consecutive immunoreaction procedures in single coronal sections.

### Epipial tracer deposits in the auditory cortex

In three ferrets, epipial neural tracer deposits were made on different regions of the auditory cortex (Fig.1B). Before surgery and to prevent postsurgical cerebral oedema, animals were given an intramuscular injection of methylprednisolone (Solu-Medrone; 10 mg/kg; Pfizer) at 1 day prior to surgery. Anaesthesia was induced with Domitor (0.022 mg/kg i.m.) and Ketaset (ketamine hydrochloride; 5 mg/kg i.m.; Fort Dodge Animal Health, Southampton, UK), and maintained with an intravenous infusion of a mixture of Domitor (0.022 mg/kg/h) and Ketaset (5 mg/kg/h) in saline. The depth of anaesthesia, respiratory rate, ECG and end-tidal CO_2_ were monitored and maintained throughout. Temperature was monitored using a rectal probe, and maintained at 38 °C using a forced-air warming system (Bair Hugger; Arizant Healthcare Inc., Eden Prairie, MN, USA). Intraoperatively, animals received methylprednisolone (20 mg/kg i.v.), Vetergesic (buprenorphine; 0.03 mg/kg s.c.; Alstoe Animal Health, York, UK) and Metacam (meloxicam; 0.2 mg/kg s.c.; Labiana Life Sciences, Terrassa, Spain) to prevent pain and inflammation, and Tagamet (cimetidine; 10 mg/kg i.v.; GlaxoSmithKline, Uxbridge, UK) to suppress stomach acid secretions. Atropine sulphate (0.06 mg/kg; AnimalCare Ltd, York, UK) was administered to reduce bronchial secretions and the eyes were protected with Viscotears (Novartis, Camberley, UK).

Animals were placed in a stereotaxic frame and a mid-sagittal incision made along the scalp. The temporal muscle on one side was retracted and the entire ectosylvian gyrus (EG) was exposed with a single craniotomy and auditory fields were mapped electrophysiologically to identify the borders between adjacent cortical regions. Single-shank *silicon* probe electrodes (Neuronexus Technologies, Ann Arbor, MI, USA) with 16 recording sites spread over a length of 1.5 mm were placed in the EG. Acoustic stimuli were generated using TDT system 3 hardware (Tucker-Davis Technologies, Alachua, FL, USA) and were presented contralaterally via a closed-field electrostatic speaker (EC1, Tucker-Davis Technologies) with a flat frequency output (± 5 dB) to ≤ 30 kHz. Closed-field calibrations of the sound-delivery system were performed using an 1/8th inch condenser microphone (Brüel and Kjær, Naerum, Denmark) placed at the end of a model ferret ear canal. Neural signals were bandpass filtered (500 Hz–3 kHz), amplified and digitized (25 kHz) using TDT System 3 digital signal processors. BrainWare software (Tucker-Davis Technologies) was used to control stimulus presentation and data acquisition, and to extract action potential clusters for analysis in Matlab (The MathWorks, Natick, MA, USA). Frequency–response areas of cortical neurons were constructed from the responses to pure-tone stimuli presented pseudorandomly at frequencies from 40 Hz to 30 kHz in one-third octave steps. Tones were 100 ms in duration (5 ms cosine ramped) and intensity levels were varied between 10 and 80 dB sound pressure level in 10 dB increments. Broadband noise bursts (40 Hz–30 kHz bandwidth and cosine ramped with a 10 ms rise/fall time, 100 ms duration from 30 to 80 dB sound pressure level) were also used as a search stimulus.

Once the different regions of the auditory cortex had been identified electrophysiologically, rhodamine (tetramethylrhodamine dextran, 3000 and 10 000 MW, lysine-fixable; Molecular Probes Inc.), fluorescein (fluorescein dextran, 3000 and 10 000 MW, anionic, lysine-fixable; Molecular Probes Inc.) and cascade blue (dextran, 10 000 MW, anionic, lysine-fixable; Molecular Probes Inc.) were applied to the middle ectosylvian gyrus (MEG), posterior ectosylvian gyrus (PEG) and anterior ectosylvian gyrus (AEG), respectively (Fig.1B). Deposits were made using sterile filter papers saturated with the 10% tracer compounds (w/v), shaped to cover the appropriate cortical region, and inserted beneath the dura mater (Rubio-Garrido *et al*., 2007). For the final rhodamine and fluorescein preparations, both 3000 and 10 000 MW solutions were mixed in equal proportions (v/v).

After tracer deposits were made, the cranium was replaced and stabilized in position with dental cement, the temporal muscles and skin were sutured independently and postoperative analgesia was provided (Vetergesic, 0.03 mL/kg i.m.; Metacam, 0.05 mL oral suspension).

Animals were perfused transcardially at 2–3 weeks after surgery as described previously. Coronal sections (35 μm thick) were prepared, and eight sets of serial sections collected. One set of sections was used for Nissl staining and another for ChAT immunocytochemistry. Three sets of sections were reserved for fluorescence microscopy and the remainder were used for rhodamine, fluorescein and cascade blue immunohistochemistry. Polyclonal rabbit anti-tetramethylrhodamine, rabbit anti-fluorescein IgG fraction and polyclonal rabbit anti-cascade blue IgG fraction (Molecular Probes Inc., Table1) were used as the primary antibodies (1 : 6000 dilution in 10 mm PBS with 0.1% Triton X_100_, 72 h at + 5 °C) after reducing non-specific binding by preincubation in 5% normal horse serum in PBS. Sections were then incubated for 2 h in the secondary antibody (biotinylated horse anti-rabbit IgG; 1 : 200 dilution; Vector Laboratories), followed by 90 min in ABC (Vector Laboratories), prior to visualization using DAB. Each step was carried out under gentle agitation and the sections were washed several times in PBS between incubations.

### Injections of the immunotoxin ME20.4-SAP in the auditory cortex

One animal received injections of the immunotoxin ME20.4-SAP (Advanced Targeting Systems) throughout the auditory cortex. This toxin comprises a monoclonal antibody specific for the p75^NTR^ membrane-bound receptor conjugated to the ribosome-inactivating enzyme saporin. Once ME20.4-SAP is bound to the external cell membranes, the saporin toxin is internalized and prevents protein synthesis, resulting in neuronal cell death (Pizzo *et al*., 1999).

The p75^NTR^ is expressed primarily by cholinergic neurons in the BF (see Results) and, following injection in the auditory cortex, is taken up only by cortical cholinergic afferents (Holley *et al*., 1994; Turchi *et al*., 2005). A microinjector (Nanoject II; Drummond Scientific Company, Broomall, PA, USA) was used to make 17 pressure injections of ME20.4-SAP at different sites in the EG at depths of 1.2 and 0.5 mm below the pial surface (ps) (Fig.1C). The volume injected at each location was 18 nL, and the total amount of toxin injected was 17 μg. Anaesthesia, perisurgical analgesia and surgical protocols were the same as before. After a survival period of 2 months, the animal was perfused and the brain processed as described for the first experimental group, using AChE histochemistry and p75^NTR^, ChAT and Pv immunocytochemistry.

### Neural tracer injections in the nucleus basalis

Rhodamine, fluorescein and biotinylated dextran amine (BDA; lysine-fixable, 10 000 MW; Molecular Probes Inc.) were dissolved in 0.9% saline (1 in 20, w/v). For the final rhodamine and fluorescein preparations, both 3000 and 10 000 MW solutions were mixed in equal proportions (v/v). Five animals received bilateral tracer injections in the NB (Fig.1D). In one case, rhodamine was injected on one side and fluorescein was injected contralaterally. In the remaining four cases, BDA was injected on the same side as rhodamine. Rhodamine and fluorescein were injected by pressure (18 nL; Nanoject II; Drummond Scientific Company), whereas BDA was administered by iontophoresis (+ 5 μA, 15 min in 7 s pulses). The tip of each glass micropipette was ≤ 20 μm external diameter and remained in place for 20 min after injections to avoid reflux of the tracer along the injection track.

Anaesthesia was induced with Domitor (0.022 mg/kg i.m.) and Ketaset (ketamine hydrochloride; 5 mg/kg i.m.), but maintained with isofluorane (IsoFlo; Abbott Laboratories Ltd, Queenborough, UK; 0.5–2.5%) in oxygen (1–1.5 L/min) after tracheal intubation. Once the animal was stabilized, medetomidine sedation was reversed with Antisedan (atipamezole; 0.5 mg/kg s.c.; Pfizer).

Stereotaxic coordinates for the NB were calculated using brain sections from an average-sized adult female ferret (700 g). Animals were placed in a stereotaxic frame and a mid-sagittal incision made along the scalp. The temporal muscles were retracted bilaterally and two craniotomies drilled 19 mm anterior to the end of the skull and 6 mm lateral to the midline. The centre of the NB is located at this anterolateral position, 8.11 mm ventral to the ps. In four cases, coordinates were adjusted by up to 200 μm medially so that two injections could be made on each side. The glass pipette was lowered slowly through a small burr hole in the skull and the dura mater to its final position, where neural tracers, described in the previous section, were injected. After the injections had been made, small pieces of Surgicel (Johnson & Johnson Medical Ltd, NJ, USA) were used to cover the exposed brain before replacing the cranium.

Animals were perfused transcardially at 2–3 weeks after surgery as described previously. Coronal sections were cut at 40 μm and seven sets of serial sections were collected in 0.1 m PB. One set of sections was stained for Nissl substance (0.5% cresyl violet) and three other series were mounted for rhodamine and fluorescein visualization using two-photon microscopy. Each of the remaining series was processed so that rhodamine, fluorescein and BDA labelling could be examined under light microscopy. Rhodamine and fluorescein were visualized using immunohistochemistry reactions, whereas BDA was detected using a Vectastain Elite ABC kit (Vector Laboratories).

### Antibody characterization

A list of the primary antibodies and their characteristics is presented in Table1. The neurofilament H non-phosphorylated (SMI_32_) mouse monoclonal antibody (Covance Research Products Inc.) recognizes only non-phosphorylated epitopes on the medium (∼ 170 kDa) and high (∼ 200 kDa) molecular weight subunits of neurofilament H in immunoblots of mammalian brain (Sternberger & Sternberger, 1983). This antibody visualizes neuronal cell bodies, dendrites, and some thick axons in the central and peripheral nervous systems, whereas other cells and tissues are unreactive (manufacturer's datasheet).

The rabbit polyclonal antibody anti-ChAT (AB143; Millipore Corporation) was prepared against human placental ChAT. By western blot, this antibody detects a single 68-kDa band representing ChAT (manufacturer's technical information). Staining of sections through the basal ganglia produced a distribution pattern of ChAT-positive neurons that was identical to previous descriptions in the monkey basal ganglia (Adams *et al*., 2001). For comparison, in some selected sections we used the ChAT antibody (ChAT rabbit polyclonal; Advanced Targeting Systems, San Diego, CA, USA), which produced the same staining pattern and the same proportion of cells as that obtained with AB143 (Millipore). The immunogen used to create the Advanced Targeting Systems antibody was a 22 amino acid peptide from porcine ChAT (GLF SSY RLP GHT QDT LVA QKSS), which was coupled to keyhole limpet hemocyanin.

The mouse monoclonal antibody to the low-affinity nerve growth factor receptor (p75^NTR^; Advanced Targeting Systems) was derived from immunization of mice with WM245 melanoma cells and recognizes p75^NTR^ in human, primate, rabbit, sheep, dog, cat, and pig. This receptor is expressed primarily by cholinergic neurons in the BF (Kordower *et al*., 1989; Tremere *et al*., 2000). According to the manufacturer's information, the antibody was tested by flow cytometry in HS294T cells.

Anti-GAD67 rabbit polyclonal antibody (Abcam plc, Cambridge, UK) has been previously validated by a 67 kDa band on western blot assays (dilution 1 : 500) in HepG2 whole-cell lysates. The antigen was a recombinant fragment, corresponding to a region within amino acids 5 and 176 of GAD67. Mouse monoclonal anti-parvalbumin (Sigma–Aldrich) is derived from the PARV-19 hybridoma produced by the fusion of mouse myeloma cells and splenocytes from an immunized mouse. Specificity was determined through western blot analysis of brain homogenate, which yields a single band at the Pv molecular weight (12 kDa) and preadsortion controls that eliminate labelling in western blots as well as cortical sections (Celio & Heizmann, 1981). This antibody labels GABAergic cells in the cortex (Celio & Heizmann, 1981) and also putative GABAergic cortical projecting neurons in the BF (Gritti *et al*., 2003).

Rabbit polyclonal IgG fraction anti-tetramethylrhodamine, anti-fluorescein and anti-cascade blue (Molecular Probes Inc.) were used as the primary antibodies. According to the manufacturer's information, these antibodies efficiently bind and quench ∼75% of the fluorescence of tetramethylrhodamine, ∼50% of that of fluorescein and ∼80% of that of cascade blue, respectively.

Negative and positive controls were always performed. No labelling was observed in sections incubated in parallel without primary or secondary antibodies. Other sections from previous experiments were always incubated in parallel to confirm consistency between antibody batches and animals. In addition, negative controls were employed when two immunoreactions were used in the same section. If double immunolabelling was performed at the same time, i.e. fluorescent ChAT and anti-p75^NTR^ co-localization, we deleted one of the second secondary antibodies (Alexa Fluor® 488 anti-rabbit or Alexa Fluor® 568 anti-mouse) in parallel control sections. When double immunoreactions were performed sequentially in the same section, i.e. p75^NTR^ and GAD67 or Pv antibodies, different enzyme detection systems were used, DAB vs. Vector VIP, and the immunoreaction was performed sequentially with the deposits of DAB over the first antigen providing a barrier that prevents the second secondary also recognizing the first primary antibody, as well as the second primary antibody. Again, further controls in single sections were employed after the first immunoreaction was finished by deleting successive steps in the second immunoreaction. A supplementary control was added when p75^NTR^ and Pv mouse monoclonal antibodies were used sequentially, with the anti-parvalbumin antibody in the second immunoreaction substituted by mouse monoclonal anti-green fluorescent protein at the same dilution (Sigma–Aldrich). In each control section, the immunostaining was similar to that produced when only the first immunoreaction was carried out, indicating that the staining in the second immunoreaction was antigen specific and not due to binding of the second primary antibody by the first secondary antibody.

### Analysis and quantification of cell bodies, fibres and terminal fields

Sections were analysed using optical and fluorescence microscopy in a Leica DMR microscope fitted with filters for fluorescence (Leica Microsystems), and a confocal microscope (LSM 710, Carl Zeiss Microimaging). The peak excitation and emission wavelengths of the fluorophores used are 402 and 421 nm for cascade blue, 490 and 525 nm for fluorescein, 490 and 525 nm for Alexa Fluor® 488, 557 and 576 nm for tetramethylrhodamine, and 578 and 603 nm for Alexa Fluor® 568 (Molecular Probes Inc.). NeuroLucida and StereoInvestigator software (MBF Bioscience, MicroBrightField Inc., Williston, VT, USA) were used for histological reconstructions and stereological estimations. Statistical analysis was performed with SPSS software (SPSS Inc., Chicago, IL, USA).

The number of ChAT- and p75^NTR^-positive cells was estimated using the fractionator principle, counting the number of positive cells in every eighth section. Positive cells were defined as those with at least two primary dendrites emerging from the cell body. A correction for uncounted sections was made according to the method used by Konigsmark (1970)
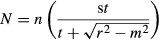
where *N* is the estimated number of positive cells, *n* is the number of cells counted, s is the number of sections, *t* is the thickness of the sections, *r* is the minimum average diameter measured in a sample of 25 positive cells, and *m* is the minimum diameter of the smallest cell in the sample. The proportions of ChAT-positive cells in the four cholinergic groups (MS, VDB, HDB and NB) were also estimated as a proportion of Nissl-stained cells in parallel adjacent sections.

The size and shape of ChAT- and p75^NTR^-positive cells were estimated by measuring a sample of 50 cells per animal in the four main subdivisions of the cholinergic BF (MS, HDB, VDB and NB). In order to have an accurate representation of all cholinergic cells throughout the BF, we sampled between one and four cells per section where at least two primary dendrites could be observed.

Fluorescent co-labelling of ChAT- and p75^NTR^-positive neurons was investigated by determining the proportion of neurons that were double or single labelled in the BF. Confocal images were captured using similar parameters of laser power, gain, pinhole and wavelengths with two channels assigned as the emission colour; *Z*-stacks were taken individually for each channel and then collapsed. Tiff images were imported directly into CorelDRAW Graphic Suite X6 (Corel Corp., Ottawa, Canada) without further editing. Co-labelling of p75^NTR^- and GAD67- or Pv-positive cells in the NB was expressed as a proportion of the total number of Nissl-stained cells in the nucleus.

The density of AChE fibres within the three regions of the auditory cortex (MEG, PEG and AEG) was analysed using the optical fractionator as a stereological probe. In the ferret auditory cortex, the SMI_32_ antibody specifically stains medium-sized pyramidal neurons in layer III and large pyramidal neurons in layer V with a distinctive pattern in the primary areas (Bajo *et al*., 2007). We therefore used the SMI_32_ staining pattern to identify the boundaries between the different regions of the EG. Estimates of the number of fibres in each region were made using the optical fractionator (Stereo Investigator software) by scanning every second or third AChE section using a square grid (0.09 mm^2^), oriented randomly, with a square counting frame of 400 μm^2^. Approximately 140 frames were counted within each section, enabling us to determine a coefficient of error and compare population sizes with 95% confidence intervals (Gundersen *et al*., 1988). The number of AChE-positive fibres in different cortical layers was estimated by the number of crossings of positive fibres over counting lines placed perpendicular to the layers at random locations in the three regions of the EG.

After checking the shape of the data distributions, the density of AChE fibres in different groups was compared using non-parametric Kruskal–Wallis tests followed, when significant differences were found, by posthoc tests for paired multiple comparisons
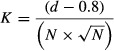
where *d* is the rank total difference between groups and *N* is the number of cases in each group. The level of significance accepted was 5%.

After making tracer injections in the NB, the labelled terminal fields in the auditory cortex were described qualitatively. The number of NB neurons labelled retrogradely by epipial tracer deposits in the auditory cortex was counted in every eighth section. When p75^NTR^ was detected in combination with Pv or GAD67 in the same section, the number of positive cells for each antibody was given as a ratio of Nissl cells in the NB.

## Results

The description of the cholinergic cells in the BF and of the AChE fibres in the ferret auditory cortex was based on data from three animals, whereas two additional cases were used to describe the co-localization of ChAT- and p75^NTR^-positive cells and putative GABAergic cells in the NB. Connections between the NB and auditory cortex were identified using epipial tracer deposits (*n* = 3) and immunotoxin injections (*n* = 1) in the auditory cortex as well as by tracer injections in the NB (*n* = 5, Fig.1).

### The ferret cholinergic basal forebrain

Cholinergic neurons were visualized using both ChAT and p75^NTR^ immunochemistry. Cholinergic neurons in the ferret BF (Ch1–4) formed a continuum that extended from the MS (Ch1), which is the most anterior and medial region, via the VDB (Ch2) and HDB (Ch3), to the most posterior and lateral structure, the NB (Ch4; Figs4). ChAT-positive neurons were more numerous than p75^NTR^-positive neurons and were more widely distributed, extending beyond the BF into the striatum [caudate nucleus (Ca), putamen and nucleus accumbens]. ChAT-positive neurons in the striatum presented a more scattered distribution than in the BF (Fig.3A and B) and, inside the striatum itself, ChAT-positive neurons were most densely packed in its ventral region (Fig.4A and B).

**Figure 2:**
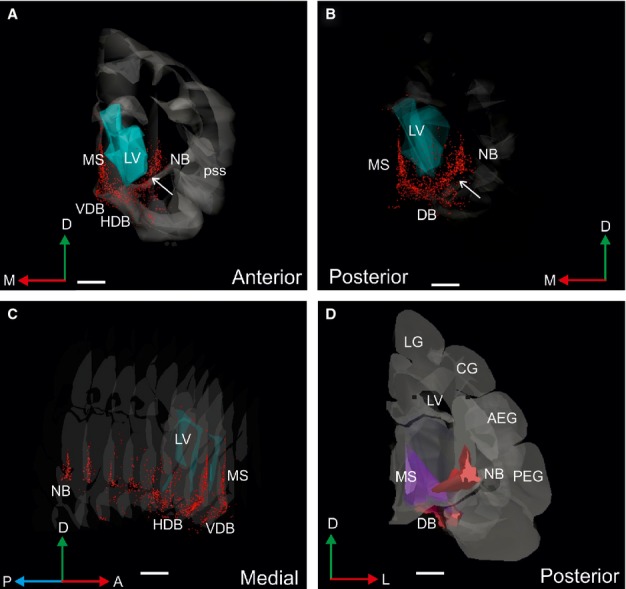
Three-dimensional reconstruction of the ferret right hemisphere showing the location of the cholinergic BF. Each red dot corresponds to a cell body in the BF immunostained with a monoclonal antibody against p75^NTR^. The image has been rotated to show anterior (A), posterior (B and D) and medial (C) views. White arrows in A and B indicate the border between the NB and HDB. (D) The NB, MS and DB are rendered as different solids. Calibration bars = 2 mm. A, anterior; AEG, anterior ectosylvian gyrus; BDA, biotinylated dextran amine; CG, coronal gyrus; D, dorsal; DB, diagonal band of Broca; HDB, diagonal band of Broca horizontal; L, lateral; LG, lateral gyrus; LV, lateral ventricle; ME20.4-SAP, monoclonal antibody specific for the p75^NTR^ conjugated to saporin; M, medial; MS, medial septum; NB, nucleus basalis; P, posterior; PEG, posterior ectosylvian gyrus; pss, pseudosylvian sulcus; sss, suprasylvian sulcus; VDB, diagonal band of Broca vertical limb.

**Figure 3:**
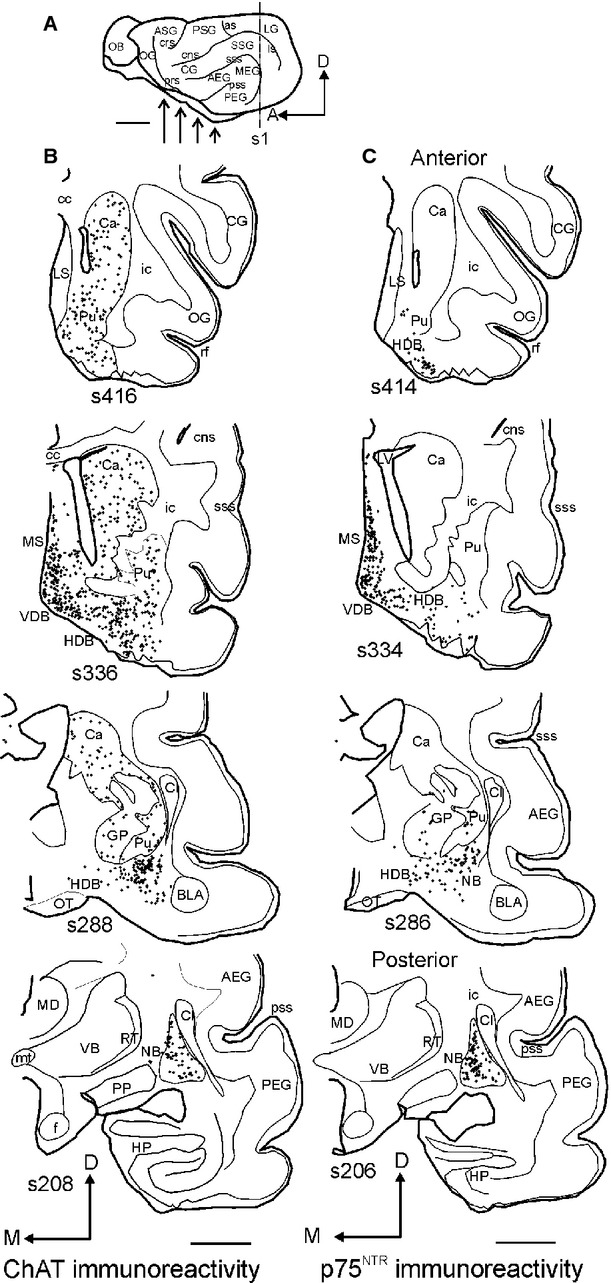
Distribution of cholinergic cells throughout the ferret BF. Examples of coronal sections taken from the right hemisphere, ordered from anterior to posterior. Each dot indicates the location of a cholinergic cell body immunostained with antibodies against ChAT (B) or p75^NTR^ (C). The region of the brain covered by these sections is represented by the arrows in A. Sections were numbered consecutively from the most posterior corner of the EG (s1 in A). Calibration bars = 5 mm in A and 2 mm in B and C. AEG, anterior ectosylvian gyrus; as, ansinate sulcus; BLA, basolateral amygdaloid nucleus; Ca, caudate nucleus; cc, corpus callosum; CG, coronal gyrus; Cl, claustrum; cns, coronal sulcus; crs, cruciate sulcus; D, dorsal; EG, ectosylvian gyrus; f, fornix; GP, globus pallidus; HDB, diagonal band of Broca horizontal limb; HP, hippocampus; ic, internal capsule; LG, lateral gyrus; LS, lateral septum; ls, lateral sulcus; LV, lateral ventricle; MD, mediodorsal nucleus of the thalamus; MEG, middle ectosylvian gyrus; MS, medial septum; mt, mammillothalamic tract; NB, nucleus basalis; OB, olfactory bulb; OG, orbital gyrus; OT, optic tract; p75^NTR^, low-affinity neurotrophin receptor; PEG, posterior ectosylvian gyrus; PP, peripeduncular nucleus; prs, presylvian sulcus; ps, pial surface; PSG, posterior sigmoid gyrus; pss, pseudosylvian sulcus; Pu, putamen nucleus; rf, rhinal ssure; RT, reticular nucleus; SMI_32_, non-phosphorylated neurolament H; SSG, suprasylvian gyrus; sss, suprasylvian sulcus; VB, ventrobasal nucleus of the thalamus; VDB, diagonal band of Broca vertical limb.

**Figure 4:**
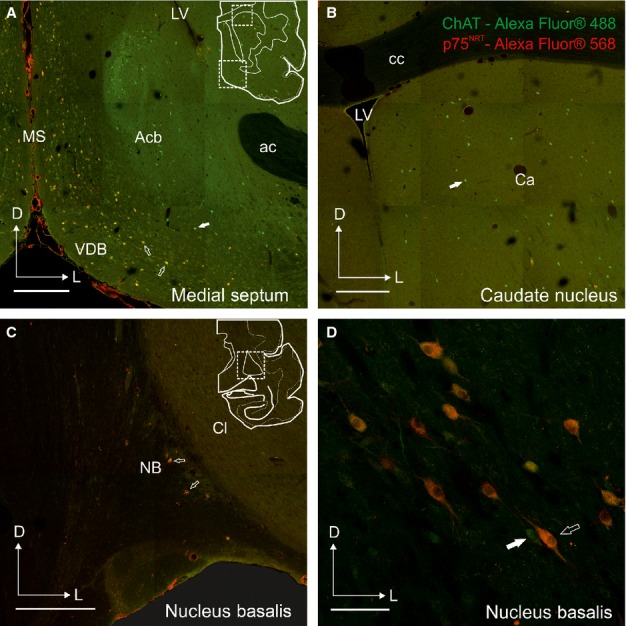
Distribution of cells co-localizing ChAT and p75^NTR^ in the ferret forebrain. Examples of coronal sections taken from the right hemisphere at the level of the MS and VDB (A), Ca (B), and more posterior at the level of the NB (C and D). The cartoons in A and C indicate the location of the photographs shown in A and B, and C and D, respectively. The majority of cells in the BF that are ChAT-positive co-localize p75^NTR^, whereas this is not the case for ChAT-immunopositive cells in the striatum. Calibration bars = 0.5 mm in A–C and 100 μm in D. ac, anterior commissure; Acb, accumbens nucleus; Ca, caudate nucleus; cc, corpus callosum; ChAT, choline acetyltransferase; Cl, claustrum; D, dorsal; L, lateral; LV, lateral ventricle; MS, medial septum; NB, nucleus basalis; p75^NTR^, low-affinity neurotrophin receptor; VDB, diagonal band of Broca vertical limb.

By contrast, p75^NTR^-positive neurons were restricted almost exclusively to the different subdivisions of the BF (Figs3 and 4). The anterior limit of the HDB extended beyond the MS with scattered ChAT-positive and p75-positive cells located between the olfactory tubercle and putamen (Fig.3B and C, s416, s414). The limit between the HDB and the NB itself was difficult to establish based on ChAT- and p75^NTR^-immunostained sections, although the neurons located ventral to the anterior commissure shared continuity with the middle part of the HDB (Fig.2).

The distribution, number and morphology of the positive elements for ChAT and p75^NTR^ in adjacent histological sections suggested that the majority of ChAT-positive cells in the BF were also p75^NTR^-immunopositive (Fig.3), which was confirmed by double immunostaining (Fig.4). In the case shown in Fig.3, the estimated number of ChAT-positive cells (Fig.3B), excluding those clearly located in the caudate/putamen, was 18 050 compared with 15 137 p75^NTR^-positive cells (Fig.3C; sampling 1 in 16 sections; 1503 ChAT-positive cells counted, a = 7.08; 1260 p75^NTR^-positive cells counted, a = 8.58). This implied that ∼ 85% of ChAT-immunopositive cells in the BF expressed the low-affinity neurotrophin receptor p75^NTR^. This percentage was even greater, up to 95%, when these counts were restricted to the NB (3854 ChAT-positive neurons vs. 3665 p75^NTR^-positive neurons). Co-localization experiments revealed the specificity of p75^NTR^ for cholinergic cells in the BF (Fig.4), whereas virtually no cells in the striatum were positive for p75^NTR^ (Fig.4A and B, solid arrows). In the MS and band of Broca, < 16% of ChAT-positive cells were p75^NTR^-negative (Fig.4A), and, in agreement with the results from single immunoreactions shown in Fig.3, most of the cells in the NB co-localized both chAT and p75^NTR^ (Fig.4C and D). Furthermore, the proportion of Nissl-stained cells that were ChAT-positive ranged from 11% in the MS and bands of Broca to 15% in the NB.

**Figure 5:**
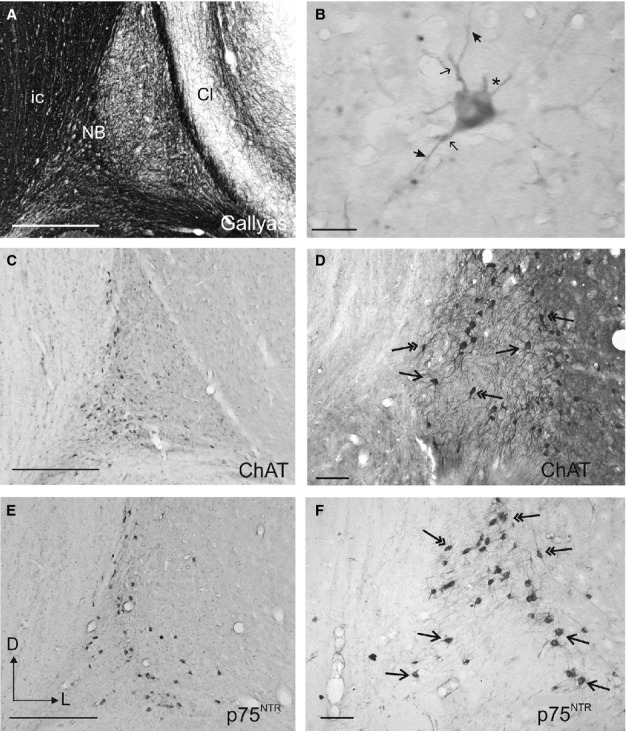
Anatomy of the NB. (A–F) Photographs taken from coronal sections at the level of the NB in the right hemisphere. Sections show myelin staining (A), ChAT immunoreactivity (C and D) and p75^NTR^ immunoreactivity (B, E and F). (B) Typical p75^NTR^-immunopositive cell in the NB. The asterisk indicates the initial part of the putative axon and the thin and thick arrows indicate the bifurcation of second-order and third-order dendrites, respectively. D and F show detail from C and E at higher magnification, respectively. Arrows and double-head arrows indicate round and elongated cells bodies, respectively. Calibration bars = 0.5 mm in A, C and E, 100 μm in D and F, and 25 μm in B. ChAT, choline acetyltransferase; Cl, claustrum; D, dorsal; ic, internal capsule; L, lateral; NB, nucleus basalis; p75^NTR^, low-affinity neurotrophin receptor.

The NB had a distinct, three-sided pyramidal shape (Figs5). The apex of this pyramid pointed dorsally, the base was oriented ventrally, and the medial face was somewhat curved (Fig.2D). Its dimensions in an average-sized female ferret (700 g) were 1.5 mm from anterior to posterior and from dorsal to ventral, and 1.3 mm mediolaterally at its broadest point (Fig.5). The HDB lay more medially and ventrally, and met the anterior part of the NB (Fig.2A and B, arrows, and Fig.2D) in the same coronal plane as the anterior limit of the pseudosylvian sulcus (pss) and the anterior corner of the PEG (Fig.3). In fact, the anteroposterior extent of the pss which subdivided the belt areas of the auditory cortex on the AEG and PEG, could be used as a surface landmark for the location of the NB. The medial limit of the NB was clearly defined by the fibres of the internal capsule (ic) (Fig.5A) and the lateral limit by the claustrum (Fig.5A), as well as by the fibres of the external capsule and the putamen at the most anterior part of the NB. Cholinergic neurons, revealed either by ChAT and/or p75^NTR^ immunochemistry, were predominantly located medially and ventrally in the cellular, less dense portion of the NB (Fig.5C and E).

The ChAT-positive and p75^NTR^-positive neurons in the NB had a multipolar morphology, with cell bodies that tended to be more globular towards the centre of the NB and more elongated in its medial and dorsal corners (Fig.5D and F, arrows and double-head arrows, respectively). Typically three to six primary dendrites emerged from each cell body, which were easier to visualize in p75^NTR^-positive cells.

Morphological values from samples of ChAT- and p75^NTR^-immunopositive neurons in the NB are shown in Table2. The form factor values of ChAT- and p75^NTR^-immunopositive neurons (0.74 ± 0.1 and 0.78 ± 0.1 for ChAT and p75^NTR^, respectively) indicated that they were far from being perfectly circular, which would mean a value of 1. They did not, however, show indentations in their soma contours, as the solidity values were close to 1 (0.97 ± 0.03 for both ChAT-positive and p75^NTR^-positive neurons, Table2). NeuroLucida reconstructions revealed no particular preferred orientation for the dendrites and a profuse dendritic arborization was suggested because of the short length of the primary dendrites (mean 16.6 μm for ChAT-positive neurons, based on 122 dendritic trees and 18.7 μm for p75^NTR^-positive neurons, based on 126 dendritic trees). In a small number of cases, fourth- and fifth-order dendrites could be observed.

**Table 2:** Cell morphological parameters from a sample of 50 ChAT- and 50 p75^NTR^-immunopositive neurons in the NB

	ChAT	p75^NTR^
Perimeter (μm)	68.33 ± 13.77	77.75 ± 11.4
Area (μm^2^)	281.64 ± 117.82	376.46 ± 119.87
Maximum diameter (μm)	27.44 ± 6.29	29.47 ± 4.82
Minimum diameter (μm)	14.47 ± 3.69	17.70 ± 4.07
Form factor (4π × area/perimeter^2^)	0.74 ± 0.1	0.78 ± 0.1
Solidity (area/convex perimeter)	0.97 ± 0.03	0.97 ± 0.03

Values are means ± SD.

A typical p75^NTR^-positive cell is shown in Fig.5B. This neuron had a polygonal cell body from which five primary dendrites emerged radially. Three primary dendrites branched into secondary dendrites (Fig.5B, thin arrows), of which two gave rise to third-order dendrites (Fig.5B, thick arrows). A thinner axon appeared to run dorsolaterally from the soma of this neuron (Fig.5B, asterisk).

### Nucleus basalis neurons that project to the auditory cortex

**Figure 6:**
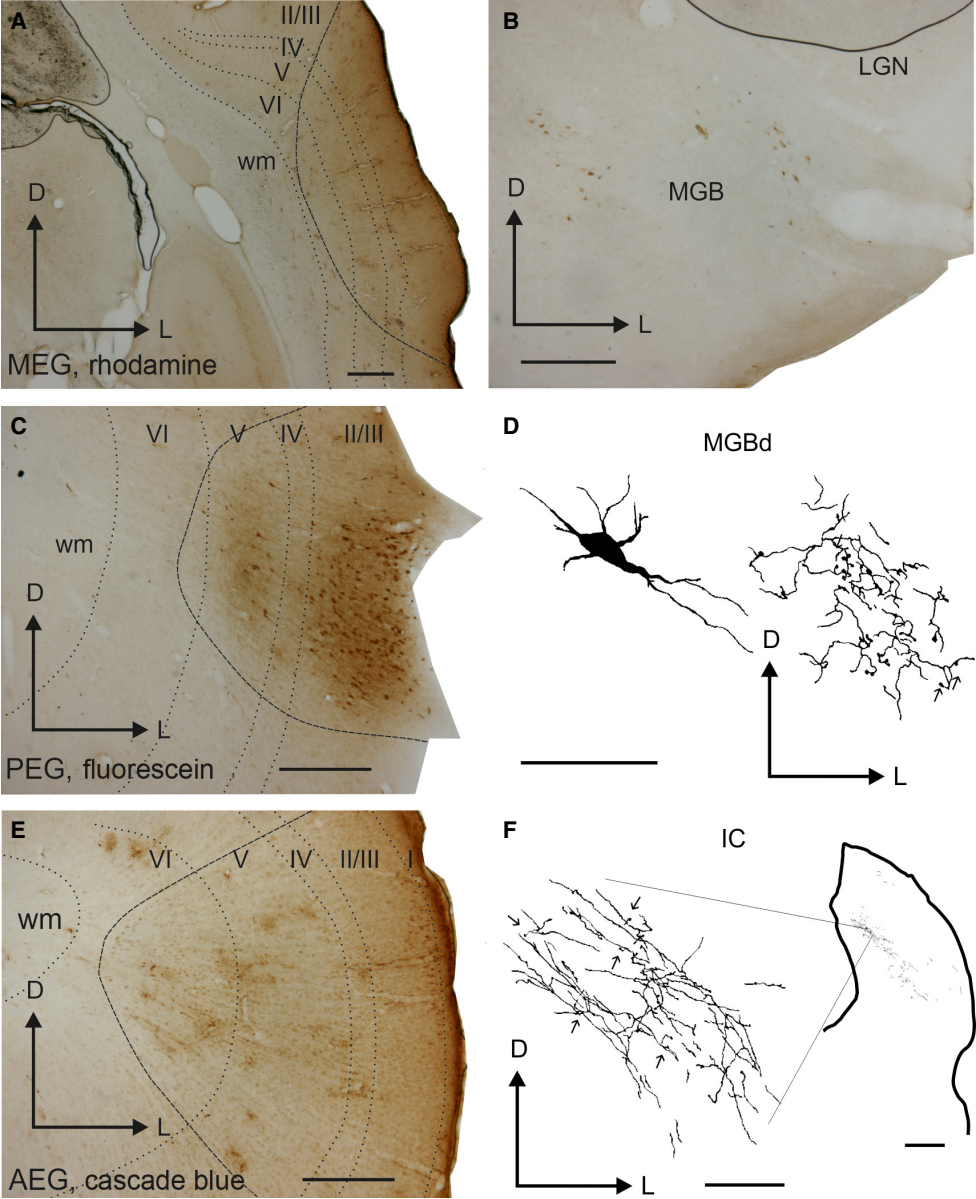
Illustration of epipial tracer deposits in the auditory cortex. Deposits of rhodamine (A), fluorescein (C), and cascade blue (E) were placed in the MEG, PEG and AEG, respectively. Labelled cells and terminal fields were observed in the MGB (B and D) and labelled terminal fields were found in the IC (F). Calibration bars = 0.5 mm in A–C and E, and 50 μm in D. In F, calibration bars for the low-magnification view of the IC and for the detail at higher magnification = 0.5 mm and 50 μm, respectively. I, II/III, IV, V, VI, cortical layers 1–6; AEG, anterior ectosylvian gyrus; D, dorsal; IC, inferior colliculus; L, lateral; LGN, lateral geniculate nucleus; MEG, middle ectosylvian gyrus; MGB, medial geniculate body; MGBd, medial geniculate body dorsal division; PEG, posterior ectosylvian gyrus; wm, white matter.

**Figure 7:**
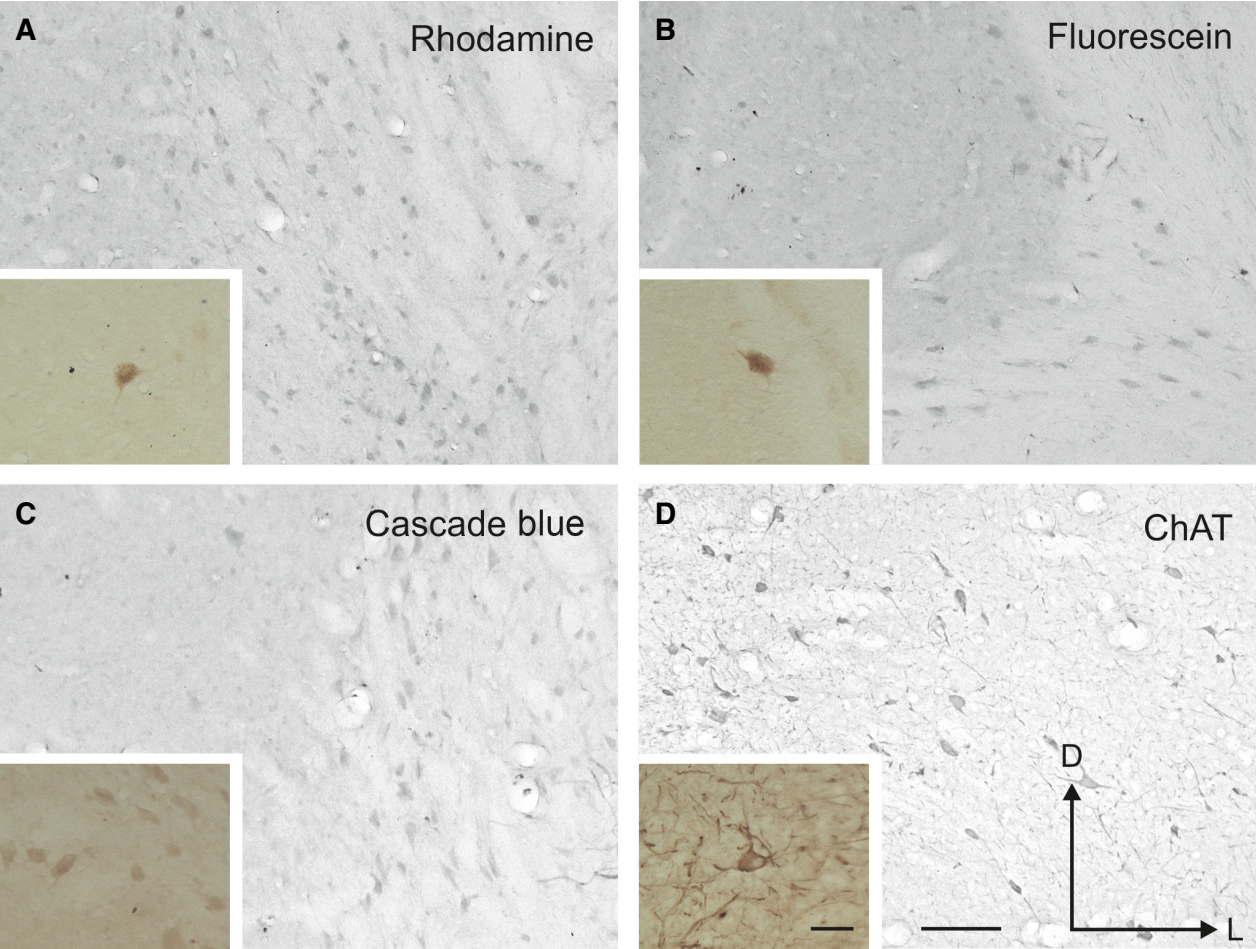
Examples of labelled cells in the NB after cortical tracer deposits of rhodamine in the MEG (A), fluorescein in the PEG (B) and cascade blue in the AEG (C). ChAT immunoreactivity in the NB is shown for comparison (D). Pictures were taken in the NB ipsilateral to the cortical tracer deposits (shown in Fig.6). The insets in the left bottom corner of each panel show labelled neurons at higher magnification. The orientation and calibration bars = 100 μm in D and 50 μm in the inset; these are the same in all panels and insets. ChAT, choline acetyltransferase; D, dorsal; L, lateral.

**Figure 8:**
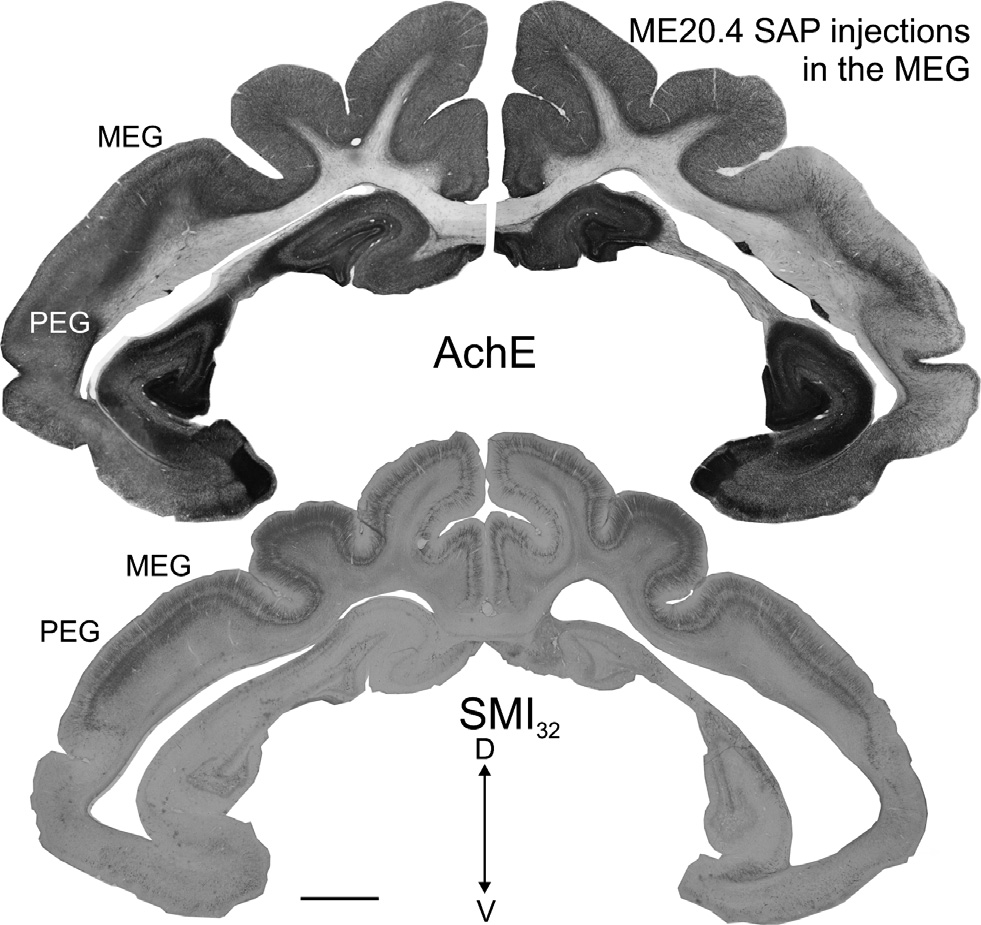
Differences in AChE staining between the two hemispheres at the level of the auditory cortex after multiple injections of the immunotoxin ME20.4-SAP in the right auditory cortex. A similar section stained with SMI32 is shown for comparison. Calibration bar = 2 mm. AChE, acetylcholinesterase; ME20.4-SAP, monoclonal antibody specific for the p75^NTR^ conjugated to saporin; MEG, middle ectosylvian gyrus; PEG, posterior ectosylvian gyrus; SMI_32_, non-phosphorylated neurofilament H; V, ventral.

**Figure 9:**
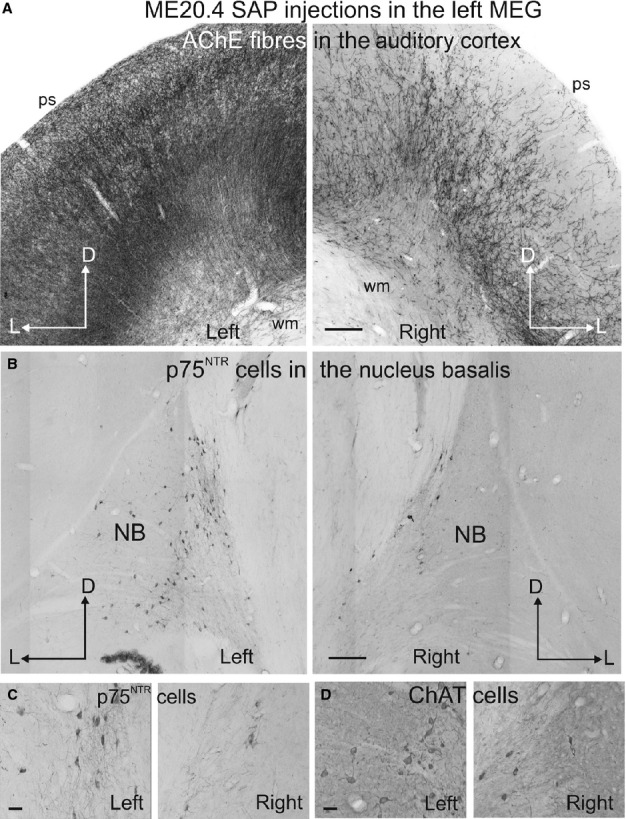
Changes in cholinergic inputs from the NB after multiple injections of the immunotoxin ME20.4-SAP in the right auditory cortex. (A) View of the left and right auditory cortex at the level of the MEG (higher magnification of the panoramic view shown in Fig.8). Low (B) and high (C and D) magnification photographs illustrating the cholinergic neurons in the left and right NB stained with an antibody against p75^NTR^ (B and C) and ChAT (D) Calibration bars = 200 μm in A and B, and 50 μm in C and D. AChE, acetylcholinesterase; ChAT, choline acetyltransferase; D, dorsal; L, lateral; p75^NTR^, low-affinity neurotrophin receptor; ME20.4-SAP, monoclonal antibody specific for the p75^NTR^ conjugated to saporin; MEG, middle ectosylvian gyrus; NB, nucleus basalis; ps, pial surface; wm, white matter.

Two approaches were used to investigate projections from the BF to the auditory cortex. First, different tracers were applied to the three regions of the EG where the auditory cortex in the ferret is located in order to retrogradely label cells in the BF (*n* = 3, Figs6 and 7). Second, multiple injections of the immunotoxin ME20.4-SAP were made in the EG (*n* = 1, Figs8 and 9) to eliminate cholinergic neurons in the BF that project to the auditory cortex. Both procedures were carried out unilaterally.

Epipial tracer deposits in the auditory cortex (Fig.6) produced light retrograde labelling in the ipsilateral NB (Fig.7), but not in the MS, VDB or HDB. No labelling was found in the contralateral BF. Tracer diffusion into the auditory cortex resulted in an inverted conical shape or “uptake cone”, the apex of which reached as far as layer V (Fig.6A, C and E). The presence of labelled cells and terminal fields in the medial geniculate body (MGB) of the thalamus (Fig.6B and D) and terminal fields in the inferior colliculus (IC) in the midbrain (Fig.6F) confirmed tracer uptake by neurons in the infragranular cortical layers. There was little diffusion of the tracer into cortical areas surrounding the filter paper. Also, after perfusion, the morphology of the cortex under the filter paper had a normal appearance apart from occasional small depressions or ruptures of the pia mater (Fig.6C).

Each of the three tracers produced specific but light retrograde labelling of neurons in the NB (Fig.7), indicating that this region of the BF projected to the whole auditory cortex. Labelled neurons were studied under brightfield and fluorescent microscopy and the measurements obtained with each were very similar. The one exception was F1023, where the intensity of the cascade blue labelling did not allow accurate cell counts to be made under brightfield illumination and so the values reported were obtained using fluorescence microscopy. Despite individual variations, the average proportion of cells labelled with each tracer indicated that the NB sent a fairly homogeneous projection to different regions of the EG (PEG deposits, 39%; MEG, 33%; AEG, 27%; Table3). The limited number of double-labelled and almost non-existent triple-labelled cells observed suggested that different subpopulations of NB neurons projected to different parts of the auditory cortex, although we found no evidence for a segregation of labelled cells within the NB based on their cortical target.

**Table 3:** Number of retrogradely labelled cells in the ipsilateral NB of 3 ferrets after making tracer deposits in different regions of the auditory cortex

	MEG (rhodamine)	PEG (fluorescein)	AEG (cascade blue)
F1014	28.6% (40/140)	41.4% (58/140)	30% (42/140)
F1023	42.5% (167/393)	44.5% (175/393)	13% (51/393)
F1026	29.2% (42/144)	32.6% (47/144)	38.2% (55/144)

The morphology of the retrogradely labelled neurons in the NB was similar to that described previously for ChAT and p75^NTR^ (compare Figs4D and 5B–F with Fig.7). These neurons were also predominantly distributed in the medial part of the NB. To confirm that the projection from the NB to auditory cortex was cholinergic, p75^NTR^-positive cells in the NB were counted after placing multiple injections of the immunotoxin ME20.4-SAP in the auditory cortex (Fig.8). These injections were made at 17 locations throughout the EG, covering both the MEG and the dorsal parts of the PEG and AEG, and resulted in a substantial loss of p75^NTR^-positive cells in the ipsilateral BF, which was largely restricted to the NB (Fig.9). The estimated numbers of p75^NTR^-positive cells in the contralateral BF (15 891) and NB (4832) of this animal were very similar to those reported earlier for normal animals, indicating that this projection was likely to be uncrossed. In contrast, the estimated number of NB p75^NTR^-positive cells ipsilateral to the injection sites (968) represented a reduction of ∼ 80% compared with the contralateral NB. The estimated numbers of p75^NTR^-positive cells in the ipsilateral and contralateral regions of the BF outside the NB were 10 345 and 11 059, respectively, indicating a reduction of only ∼ 6% of the neurons in these regions. This dramatic reduction in the NB ipsilateral to the immunotoxin injections could easily be seen at the level of individual sections (Fig.9B).

In agreement with the substantial loss of p75^NTR^-positive neurons in the ipsilateral NB, the density of AChE-positive fibres in the auditory cortex on that side was similarly reduced relative to the contralateral side. This was particularly the case in the MEG and in layer III (Figs8 and 9A). Optical fractionator estimates revealed that the AChE fibre density in the contralateral cortex was similar to that found in normal ferrets (see Fig. 4), with density values of 0.76 for the MEG, 1.06 for the PEG and 0.84 for the AEG (expressed in units of thousands/μm^3^). The AChE fibre density in the ipsilateral auditory cortex was, however, reduced by nearly 60% (density values of 0.22 for the MEG, 0.55 for the PEG, and 0.37 for the AEG). As expected from the location of the immunotoxin injection sites, the largest difference was seen in the MEG.

No signs of damage were observed in the auditory cortex where the immunotoxin was injected (Figs8 and 9A). Furthermore, to rule out the possibility that non-specific cell death, resulting from axotomy due to the multiple injection sites, was responsible for the decrease in AChE density in the cortex or of cholinergic neurons in the NB, the number of cells in the auditory thalamus was estimated on both sides using the optical fractionator. Three Nissl-stained sections taken at the centre of the MGB were selected. After tracing the limits of the MGB, the total number of neurons on both sides was estimated using counting frames of 250 μm^2^ in a 0.4 mm^2^ grid. Very similar cell densities were found in the MGB both ipsilateral and contralateral to the cortical injections (3.64 vs. 3.61 cell bodies/mm^3^, respectively), indicating that the immunotoxin injections had not caused a loss of thalamocortical projection neurons.

**Figure 10:**
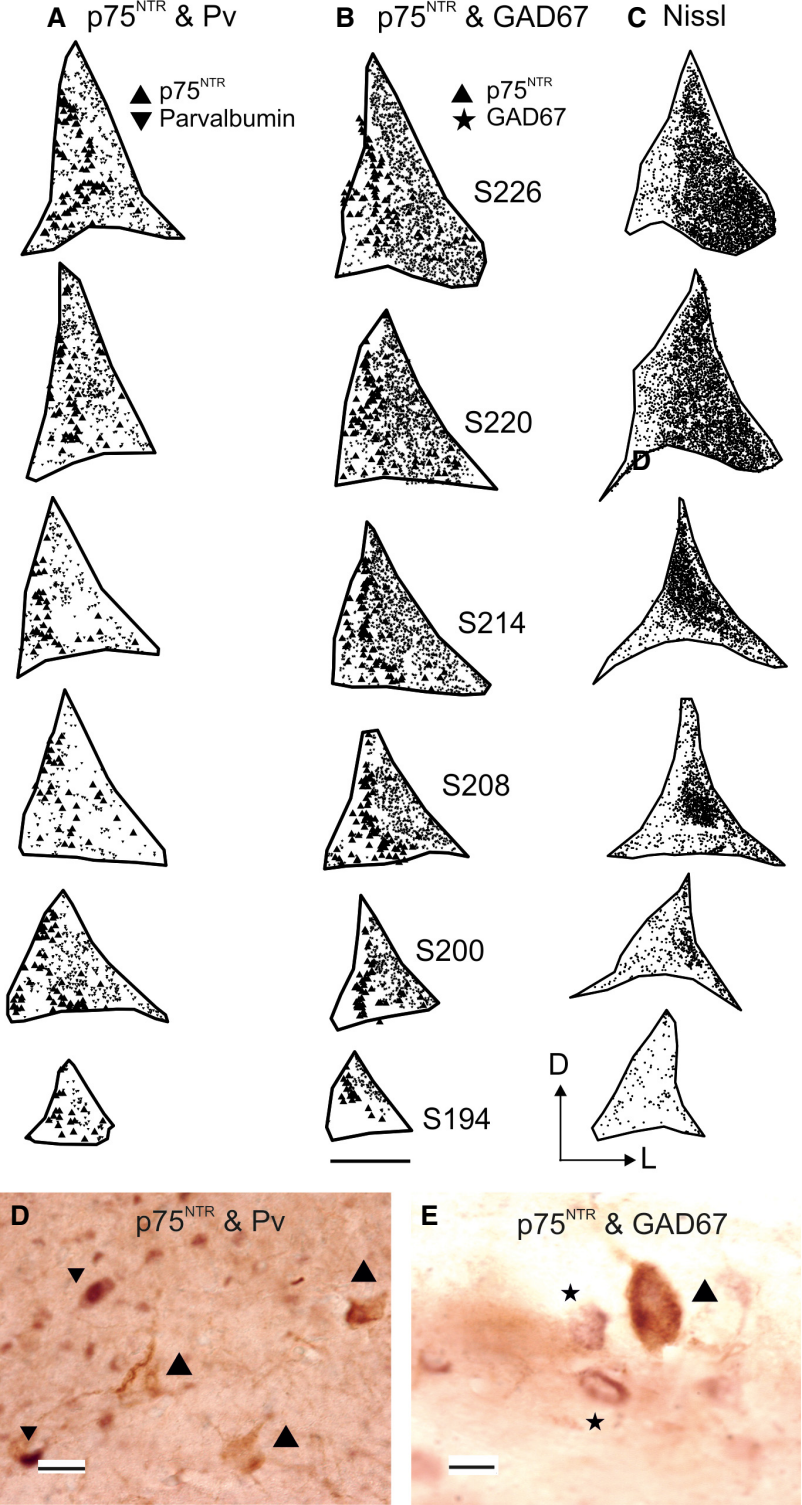
Distribution of p75^NTR^-, Pv- and GAD67-immunoreactive-positive cells in the NB. Coronal sections (1/6th sample) are taken from the right hemisphere and ordered from anterior to posterior. Sections in B are numbered consecutively beginning at the level of the posterior corner of the EG (Fig.3A); sections shown in A and C are adjacent to those in B. (A) Distribution of p75^NTR^-positive and Pv-positive (large and small symbols, respectively) cells in the NB. (B) Distribution of p75^NTR^-positive and GAD67-positive (large and small symbols, respectively) cells. (C) Total number of cells stained for Nissl substance. Examples of labelling for both p75^NTR^ and Pv, and for p75^NTR^ and GAD67 are shown in D and E, respectively. P75^NTR^-positive cells are indicated by large point-up triangles, Pv-positive cells by small point-down triangles and GAD67-positive cells by asterisks. Calibration bars = 1 mm in A–C, 100 μm in D and 50 μm in E. D, dorsal; EG, ectosylvian gyrus; GAD67, 67 kDa glutamic acid decarboxylase; L, lateral; p75^NTR^, low-affinity neurotrophin receptor; Pv, parvalbumin.

As non-cholinergic cells in the NB also project to the sensory cortices (Gritti *et al*., 2003; Hur & Zaborszky, 2005), we explored the distributions of the GAD67- and Pv-immunopositive cells and compared these with that of p75^NTR^-positive neurons in the NB (Fig.10). No neurons were double-labelled for p75^NTR^ and Pv or GAD67. The distribution of cholinergic (p75^NTR^-positive) and GABAergic (GAD67-positive and Pv-positive) cells indicated that the two subpopulations only partially overlapped, with the inhibitory cells located more lateral and ventral in the NB (Fig.10), where the cell density was higher (Fig.10C). GAD67-positive cells constituted almost half of the neurons in the NB (45% of the proportion of Nissl-stained cells measured in alternate contiguous sections), whereas Pv-positive and p75^NTR^-positive cells, respectively, made up 15 and 3% of the neurons found there.

### Nucleus basalis inputs to the auditory cortex

**Figure 11:**
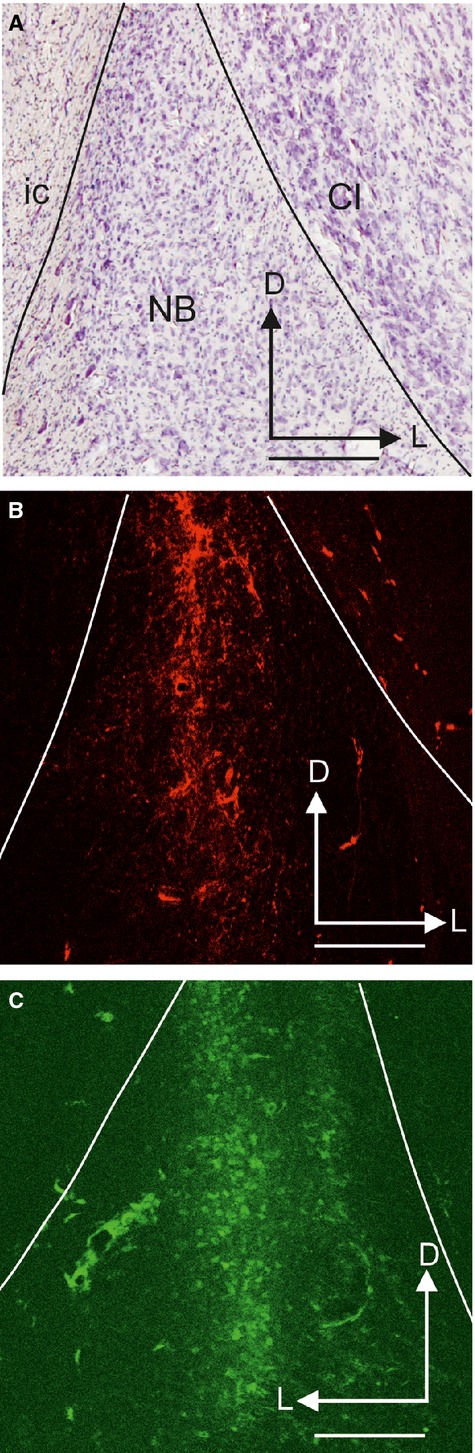
Examples of fluorescent tracer injection sites in the NB in a single animal. (A) Location and limits of the right NB in a Nissl-counterstained section. (B) Rhodamine injection site in the right NB. (C) Fluorescein injection site in the left NB (B and C visualized using a two-photon microscope). Calibration bars = 250 μm. Cl, claustrum; D, dorsal; ic, internal capsule; L, lateral; NB, nucleus basalis.

Tracer injections in the NB always resulted in labelled terminals in the auditory cortex. Typical examples of injection sites are illustrated in Fig.11, which shows a single rhodamine injection site in the right NB (Fig.11B) and two fluorescein injection sites in the left NB (Fig.11C).The glass pipette was advanced dorsoventrally in order to minimize the risk of encroaching on the substantial fibre tracts that surrounded the NB both laterally and medially.

The pattern of labelled fibres and terminals in the auditory cortex (Figs2 and 3) resembled that observed in sections stained for AChE (Fig.4, see also Figs8 and 9A), although the density of labelling was much less than that of AChE-stained fibres. Labelled fibres were observed throughout all regions of the auditory cortex, and, as with the AChE staining, layers II/III and the infragranular layers were the most densely labelled (Figs4 and 5). Labelled fibres were predominantly oriented parallel to the cortical layers, particularly in the infragranular layers (Fig.3B), whereas the orientation of the terminal fields in layers II/III was more trabecular (Fig.3A). Other fibres were oriented perpendicular to the cortical layers with short collaterals along their main trajectory that ran parallel to the layers, and that contained one or two *en-passant* varicosities and ended in a single terminal swelling (Fig.3C). *En-passant* varicosities and terminal labelled swellings were always small and round (≤ 1–2 μm in size) and usually had single endings (Fig.3A–C, arrows and asterisks, respectively), a pattern that was also observed in other areas of the cortex, including the orbital gyrus in the frontal cortex (not shown). Large or complex terminals in the cortex were not observed after tracer injections in the NB.

**Fig 12:**
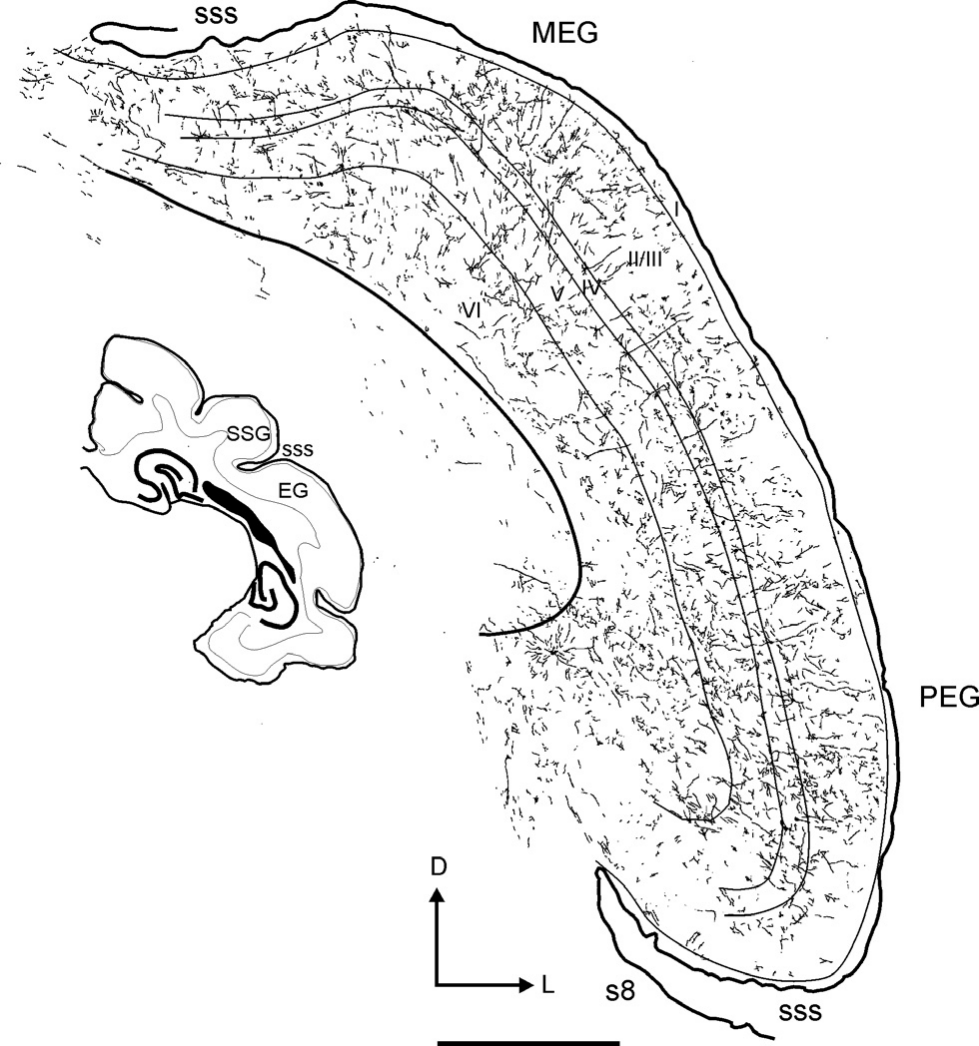
Drawing of a representative section at the level of the auditory cortex after injection of rhodamine in the ipsilateral NB. The distribution of labelled terminal fields and neurons across the different cortical layers is shown. The inset shows a low-magnification view of this section. Calibration bar = 1 mm. I, II/III, IV, V, VI, cortical layers 1–966; D, dorsal; EG, ectosylvian gyrus; L, lateral; MEG, middle ectosylvian gyrus; PEG, posterior ectosylvian gyrus; SSG, suprasylvian gyrus; sss, suprasylvian sulcus.

**Figure 13:**
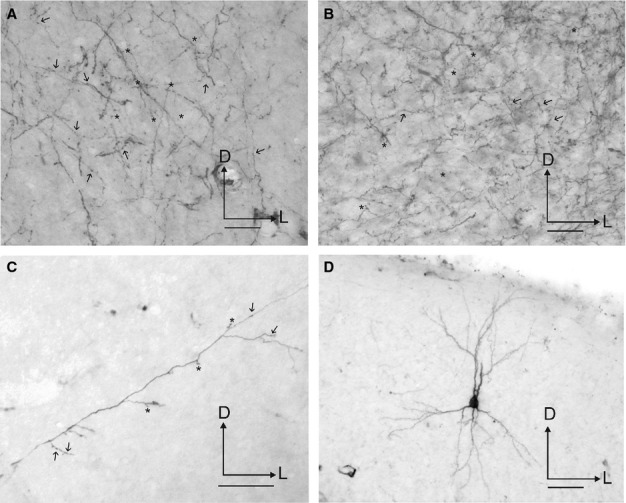
Photographs of labelling in the auditory cortex after tracer injections in the NB. Terminal fields are shown in the supragranular (A) and infragranular (B) layers at the level of A1. (C) Example of an individual fibre crossing perpendicular to the cortical layers. (D) A retrogradely labelled neuron in A1. Arrows and asterisks indicate examples of *en-passant* varicosities and terminal swellings, respectively. Orientation of the sections is the same in every picture and is the same as in Fig.12. Calibration bars = 25 μm in A–C, and 50 μm in D. D, dorsal; L, lateral.

**Figure 14:**
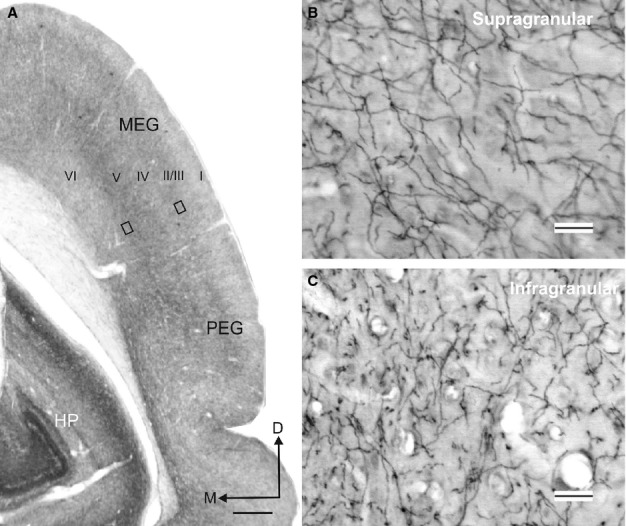
Acetylcholinesterase (AChE) staining in the auditory cortex. (A) Coronal section at the level of the auditory cortex in the EG illustrating the variation in AChE fibre density across the cortical layers. (B and C) Details from the two rectangles in A taken at higher magnification to show AChE-positive fibres in the supragranular and infragranular layers of the cortex, respectively. Calibration bars = 1 mm in A and 25 μm in B and C. I, II/III, IV, V, VI, cortical layers 1–6; D, dorsal; EG, ectosylvian gyrus; HP, hippocampus; M, medial; MEG, middle ectosylvian gyrus; PEG, posterior ectosylvian gyrus.

**Figure 15:**
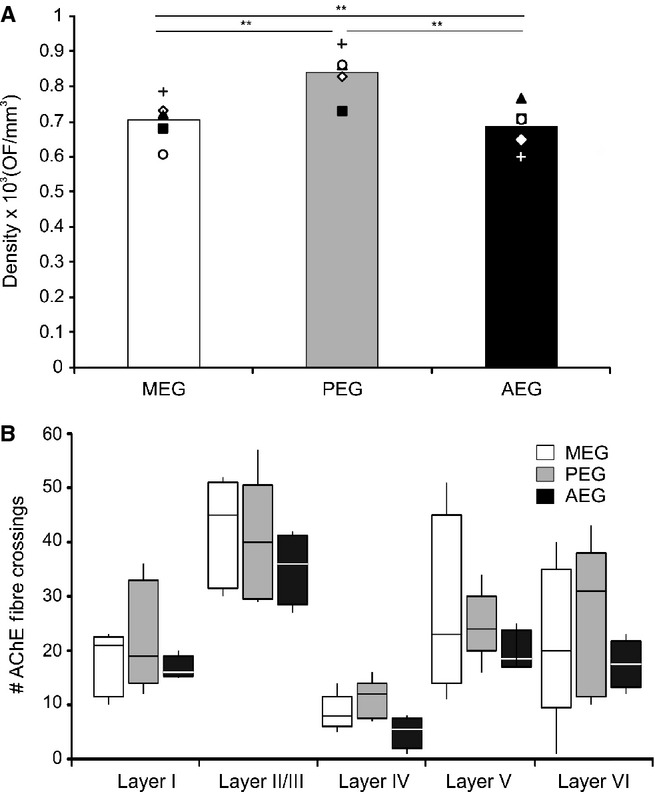
Quantification of AChE fibre density by cortical region (A) and layer (B). (A) Density values, estimated using an optical fractionator (OF) stereological probe, from five individual hemispheres are plotted as symbols and their mean as the histogram bar. (B) Medians, 25th and 75th percentiles, and ranges are plotted by region and layer, taking layers II and III together. The white bars indicate values for the MEG, grey bars for the PEG and black bars for the AEG. Statistical significance: **P =* *0.01. I, II/III, IV, V, VI, cortical layers 1–6; AChE, acetylcholinesterase; AEG, anterior ectosylvian gyrus; MEG, middle ectosylvian gyrus; PEG, posterior ectosylvian gyrus.

Retrogradely labelled neurons were also observed in layers II/III and V of the auditory cortex, especially in the dorsal part of the MEG and AEG close to the suprasylvian sulcus, and in the neighbouring suprasylvian gyrus (not shown). These neurons were pyramidal in shape, with both the cell body and most of the dendritic tree heavily labelled (Fig.3D), suggesting tracer uptake by fibres of passage. Even with injection sites located within the NB, as in Fig.1, the possibility of tracer uptake by surrounding fibres could not be excluded. Interestingly, when injection sites were located more medially, a larger number of layer V pyramidal cells were labelled in the auditory cortex, whereas labelled terminal fields were also observed in the MGB. More lateral injection sites, however, predominantly labelled pyramidal cells in layers III and V.

Due to the high density of the labelled terminal fields and the presence of some retrograde labelling in the auditory cortex, quantification of the distribution and numbers of putative boutons was not performed. However, the number and distribution of terminal swellings and *en-passant* varicosities appeared to be very similar (Fig.3A–C, arrows and asterisks, respectively). The pattern of labelled fibres and terminals in the auditory cortex resembled that observed in sections stained for AChE (Fig.4). AChE fibre staining in the auditory cortex was particularly strong in layers II/III and V (Fig.4A; see also Figs8 and 9A). In the supragranular cortical layers, AChE fibres had a trabecular pattern and were oriented both parallel and perpendicular to the layers (Fig.4B), whereas in the infragranular layers they were often cut in cross section, indicating a more oblique anteroposterior orientation (Fig.4C).

Estimation of the AChE fibre density in the EG by the optical fractionator revealed no differences between individual animals or between the left and right hemispheres (Fig.5). The fibre density did vary, however, across different regions of the auditory cortex (Fig.5A; non-parametric Kruskal–Wallis test, *F*_2,11_ = 7.46, *P *=* *0.009), with the heaviest staining in the PEG [MEG, 0.71 ± 0.067; PEG, 0.84 ± 0.071; AEG, 0.67 ± 0.068 (expressed in units of thousands/μm^3^, mean ± SD)]. Significant differences in density were found between every region considered (Kruskal–Wallis sample posthoc test for paired comparisons – MEG/PEG, *K* = 12.16, *P *=* *0.01; MEG/AEG, *K* = 4.11, *P *=* *0.01; PEG/AEG, *K* = 16.64, *P *=* *0.01).

The quantification of AChE staining in different cortical layers was carried out on a small number of samples (20 per region) by counting the number of fibres that crossed the reference lines placed perpendicular to the cortical layers. Significant differences in fibre numbers across layers were found within each of the three subdivisions of the EG (Kruskal–Wallis test – MEG, *F*_1,47_ = 85.003, *P *<* *0.01; PEG – *F*_1,47_ = 147.35, *P *<* *0.01; AEG – *F*_1,37_ = 60.00, *P *<* *0.01). In all areas, layers II/III were the most heavily stained and layer IV had the lightest staining (Figs4A and 5B).

## Discussion

The present experiments describe the architecture of the ferret cholinergic BF and its projections to the auditory cortex. Our results demonstrate that ipsilateral projections from the NB provide the main source of cholinergic input to the auditory cortex in this species and that these inputs are not homogeneously distributed across different cortical regions or layers. Moreover, we show that, as in other species, the ME20.4-SAP immunotoxin provides an effective method for selectively eliminating cholinergic neurons that project from the NB to the auditory cortex in ferrets, demonstrating that this approach can be used to investigate the role of cholinergic modulation in cortical processing and plasticity.

### The cholinergic basal forebrain in the ferret

In mammals, the BF comprises four main cholinergic groups – the MS (Ch1), VDB and HDB (Ch2 and 3, respectively) and NB (Ch4; Henderson, 1987a; Woolf, 1991). We used the low-affinity neurotrophin receptor p75^NTR^ as a marker for cholinergic neurons in the ferret BF because of its known specificity for such neurons in other species (Tremere *et al*., 2000) and the high degree of co-localization reported between p75^NTR^ and the specific cholinergic marker ChAT (Kordower *et al*., 1989). Our results confirm the distribution of cholinergic cells previously described in the ferret BF by Henderson (1987a), and show that ∼ 85–95% of ChAT-positive cells in the BF express p75^NTR^. Double-immunostaining experiments revealed that ChAT- and p75^NTR^-immunopositive neurons share the same distribution within the NB and are similar in their morphology. The small differences in size observed between ChAT-positive and p75^NTR^-positive neurons are likely to be due to differences in the immunostaining protocols used and, in particular, to the inclusion of the detergent Triton X_100_ in our primary antibody incubation stage for ChAT.

Our three-dimensional reconstruction of the distribution of neurons expressing p75^NTR^ demonstrates that these cells form a continuum through the ferret BF, whereas epipial tracer deposits and immunotoxin injections in the EG show that BF neurons that project to the auditory cortex arise almost exclusively in the ipsilateral NB. As ME20.4-SAP is taken up only at cortical terminals that express p75^NTR^, the 80% reduction of this cell type in the ipsilateral, but not contralateral, NB further confirms that neurons expressing p75^NTR^ provide the majority of the cholinergic input to the auditory cortex.

Using targeted injections of horseradish peroxidase, Henderson (1987b) reported that the densest inputs to the ferret visual cortex arise in the most posterior regions of the NB, similar to the pattern of cholinergic innervation observed in the primate striate cortex (Mesulam *et al*., 1983). However, unlike primates, there is some evidence that the DB provides a small proportion of cortical cholinergic input in the ferret (Henderson, 1987b). This is consistent with the very modest reduction observed in the present study in the number of p75^NTR^-positive cells in regions of the BF outside the NB following injections of immunotoxin in the ipsilateral auditory cortex. The rat cortex, however, receives a much higher proportion of its cholinergic input from the DB, although the majority of projections to sensory cortex still originate in the NB (Henderson, 1981).

The proportion of cortical cholinergic afferents provided by the NB in ferrets appears therefore to lie somewhere between that observed in primates, whose inputs are restricted to the NB alone, and rodents, where cholinergic neurons that project to the cortex are more widely distributed in the BF. Kamke *et al*. (2005a) combined wheatgerm agglutinin-conjugated horseradish peroxidase retrograde tracer injections in A1 with ChAT immunocytochemistry in cats, and found that the majority of the double-labelled neurons were located in the lateral BF, including the globus pallidus, putamen and ic, with very little labelling in the DB or MS. Thus, it is clear that functional investigations into the role of cholinergic modulation in auditory cortical function in primates and carnivores, such as the ferret, should focus on the NB.

Not all cholinergic BF neurons express p75^NTR^, which probably accounts for the lower cell counts for p75^NTR^-positive neurons compared with ChAT-positive neurons. In the rat, at least some of the cholinergic neurons without p75^NTR^ project to the amygdala (e.g. Hecker & Mesulam, 1994). It is not known whether such neurons project to the auditory cortex. For this reason, p75^NTR^ immunocytochemistry may slightly underestimate the number of corticopetal cholinergic neurons.

### Cholinergic inputs to the auditory cortex

Although AChE histochemistry is not a proxy for cholinergic fibres and can stain other cell types, including dopaminergic fibres (Mizukawa *et al*., 1986), the distribution of AChE fibre staining has been shown to match that of ChAT-immunopositive fibres in the rat auditory cortex (Lysakowski *et al*., 1989). We found that the AChE staining pattern in the ferret auditory cortex closely resembles that of the fibres and terminal fields obtained after making tracer injections in the NB (compare Figs12 and 14). Moreover, lesioning NB cholinergic neurons results in a substantial reduction in AChE cortical staining in ferrets (Figs8 and 9A; see also Leach *et al*., 2013) and cats (Kamke *et al*., 2005a,b), indicating that many of the AChE cortical fibres are in fact cholinergic.

Differences in the laminar distribution of AChE fibres have been reported between cortical regions and species (Mesulam *et al*., 1984; Henderson, 1987a; Wallace *et al*., 1991; Kamke *et al*., 2005a; Anderson *et al*., 2009). In primates, Mesulam *et al*. (1984) showed that AChE staining in the visual, auditory and somatosensory cortex is particularly heavy in layers III/IV, whereas motor and premotor areas are characterized by dense concentrations of radially-arranged fibres in the deepest layers of the cortex. In the mouse, Anderson *et al*. (2009) reported that AChE staining is strongest in layer IV and lower layer V in the visual and somatosensory cortex, but distributed more homogeneously across the layers of the auditory cortex. We found that AChE staining density peaked in layer II/III and in the infragranular layers in all regions of the ferret auditory cortex. Because we examined sections that were cut in the coronal plane only, it is possible that our measurements might have been biased due to differences in fibre orientation between the supragranular and infragranular layers, which, in turn, might explain the high variability obtained in the latter (Fig.5). However, the laminar pattern of AChE staining that we observed in the ferret auditory cortex is similar to that reported for the cat A1 (Wallace *et al*., 1991; Kamke *et al*., 2005a). Thus, cholinergic influences on the response properties of these neurons would be expected to vary across different layers of the cortex.

Injections of tracers into the NB produced extensive anterograde labelling in the auditory cortex. Some retrogradely labelled cell bodies were also observed in layers III and V of the cortex. However, epipial tracer deposits in the EG produced virtually no labelled terminals in the NB, even when those tracers were taken up by neurons in the infragranular cortical layers, as demonstrated by the presence of both labelled neurons and terminals in the MGB and labelled terminals in the IC. It is therefore likely that retrograde labelling of pyramidal cells in the auditory cortex following tracer injections in the NB reflected tracer uptake by the fibre tracts that surround this nucleus. Indeed, there is always the possibility of some tracer uptake by axons of the ic medially and the external capsule laterally when injections are made into the NB, which is the main reason why we also made tracer deposits in the auditory cortex in order to characterize the connectivity of these regions.

Although the NB neurons that project to the ferret auditory cortex closely resembled both ChAT- and p75^NTR^-immunopositive cells in their morphology and distribution, non-cholinergic projection neurons have been described in many other species, including carnivores (Fisher *et al*., 1988). Consequently, it is likely that some of the neurons retrogradely labelled in the NB after epipial tracer deposits in the auditory cortex were non-cholinergic. Non-cholinergic corticopetal BF neurons are predominantly GABAergic and may contain various calcium-binding proteins, including Pv and neuropeptide Y (Duque *et al*., 2000, 2007; Gritti *et al*., 2003; Zaborszky *et al*., 2005). These putative inhibitory neurons project to the cortex (Fisher *et al*., 1988; Gritti *et al*., 1997, 2003) and also form local connections within the BF itself (Duque *et al*., 2007). Another set of non-cholinergic, non-GABAergic projection neurons have been described in the BF that may use glutamate as a neurotransmitter (reviewed in Semba, 2000), although, according to Hur & Zaborszky (2005), the proportion of corticopetal BF neurons co-localizing vesicular glutamate transporter (VGluT2) is very small.

### Cholinergic influences on auditory cortical processing

The widespread distribution of afferents from the NB to the auditory cortex suggests that this region of the BF is likely to be involved in modulating many aspects of cortical processing. Several auditory cortical fields have been characterized functionally in the ferret EG using intrinsic optical imaging (Nelken *et al*., 2004) and electrophysiological measurements (Bizley *et al*., 2005). Differences in sensitivity to spatial and non-spatial sound features have been identified between these cortical fields (Bizley *et al*., 2009; Walker *et al*., 2011), and they also vary in the extent to which they receive inputs from other sensory modalities (Bizley *et al*., 2007; Mao *et al*., 2011). Moreover, somatosensory and visual areas have also been described in the surrounding pseudosylvian and suprasylvian sulcal regions, which may extend onto the EG itself (e.g. Ramsay & Meredith, 2004; Manger *et al*., 2005; Keniston *et al*., 2009; Homman-Ludiye *et al*., 2010). The presence of labelled terminals in these locations, as well as all three main regions of the EG, after placing tracer injections in the NB suggests that cholinergic inputs are likely to affect both auditory and multisensory functions.

We observed few double-labelled and only very occasional triple-labelled neurons in the NB after epipial tracer deposits, implying that different regions of the auditory cortex are likely to be innervated by different BF neurons. This is consistent with descriptions of cholinergic BF neurons projecting to the cortex having limited axon collaterals (Duque *et al*., 2007) and with the small proportion of double-labelled cells found in the BF after tracer injections in the cortex (Semba, 2000). The different cell types in the BF show regionally-specific distributions (Zaborszky *et al*., 2005), whereas more recent work suggests that cholinergic and non-cholinergic neurons that project to the neocortex comprise distinct populations that vary in the extent to which they overlap according to the degree of connectivity between their cortical targets (Zaborszky *et al*., 2013). Collectively, these findings suggest that distinct pools of NB neurons could potentially convey signals from the limbic system about the emotional significance of stimuli (Mesulam & Mufson, 1984) or from the prefrontal cortex about their behavioural salience (Golmayo *et al*., 2003; Rasmusson *et al*., 2007) in parallel to different regions of the neocortex.

The cholinergic modulation of cortical neurons has been implicated in various cognitive functions, including attention, learning and memory (Weinberger, 2003; Hasselmo & Sarter, 2011; Klinkenberg *et al*., 2011; Edeline, 2012). In particular, there is considerable evidence that cholinergic inputs to the auditory cortex are involved in learning and memory. This is largely based on the receptive field plasticity induced in the A1 by repeatedly pairing tones with NB stimulation (e.g. Kilgard & Merzenich, 1998a; Weinberger & Bakin, 1998; Ma & Suga, 2003; Froemke *et al*., 2007). Cortical application of atropine, a muscarinic receptor antagonist, prevents this reorganization (Froemke *et al*., 2007) as well as the cortical plasticity that results from conditioning (Ji *et al*., 2001), confirming a specific role for cholinergic inputs in these changes.

Cholinergic inputs are also likely to be involved in mediating plasticity in non-primary auditory cortical areas. Thus, repeated pairing of tones and NB stimulation can also reorganize tonotopic map representations in the posterior auditory field of the rat (Puckett *et al*., 2007), although the nature of the frequency-specific plasticity observed within individual animals differs between primary and secondary auditory regions. The integrity of all three regions of the EG is required for the training-induced plasticity of auditory localization that has been demonstrated in adult ferrets (Nodal *et al*., 2010, 2012). As our anatomical data suggest that the entire auditory cortex can be influenced by cholinergic inputs, the finding that ME20.4-SAP immunotoxin lesions of the NB impair this cortex-dependent spatial learning (Leach *et al*., 2013) could potentially reflect a loss of cholinergic modulation in any of these cortical regions.

Rapid changes in the response properties of A1 neurons take place when ferrets are engaged in behavioural tasks that require them to pick out a target sound against a background of other sounds (Fritz *et al*., 2007; Atiani *et al*., 2009). Related effects have recently been described in the frontal cortex of this species, which may contribute to the task-dependent plasticity of A1 neurons (Fritz *et al*., 2010). In rats, prefrontal cortex inactivation abolishes stimulus-evoked release of acetylcholine in sensory areas of the cortex (Rasmusson *et al*., 2007), suggesting a circuit by which top-down signals can mediate ongoing task demands via cholinergic modulation of the responsiveness and tuning properties of sensory cortical neurons.

The BF is not the only source of cholinergic input to the auditory system. Cholinergic circuits also arise in the pontomesencephalic tegmentum (cholinergic groups 5 and 6) in the midbrain. However, Henderson (1987b) found no discernible cholinergic inputs from the midbrain to the visual cortex, indicating that cortical acetylcholine is likely to be provided entirely by the cholinergic projections from the BF. Nevertheless, the pontomesencephalic tegmentum innervates the MGB and IC among other regions (Schofield *et al*., 2011), providing a subcortical route by which acetylcholine can induce stimulus-specific plasticity (Luo *et al*., 2011). Further work is needed to determine the relationship between forebrain and midbrain cholinergic circuits and their relative contributions to central auditory processing and learning.

## Abbreviations

I, II/III, IV, V, VI: cortical layers 1–6
A1: primary auditory cortex
AChE: acetylcholinesterase
AEG: anterior ectosylvian gyrus
BDA: biotinylated dextran amine
BF: basal forebrain
ChAT: choline acetyltransferase
DAB: 3,3′- diaminobenzidine tetrahydrochloride
DB: diagonal band of Broca
EG: ectosylvian gyrus
GAD67: 67 kDa glutamic acid decarboxylase
HDB: diagonal band of Broca horizontal limb
IC: inferior colliculus
ME20.4-SAP: monoclonal antibody specific for the p75^NTR^ conjugated to saporin
MEG: middle ectosylvian gyrus
MGB: medial geniculate body
MS: medial septum
NB: nucleus basalis
PBS: phosphate-buffered saline
PEG: posterior ectosylvian gyrus
Pv: parvalbumin
SMI_32_: non-phosphorylated neurofilament H
VDB: diagonal band of Broca vertical limb
